# Structural and kinetic characterization of DUSP5 with a Di-phosphorylated tripeptide substrate from the ERK activation loop

**DOI:** 10.3389/fchbi.2024.1385560

**Published:** 2024-08-05

**Authors:** Andrea Imhoff, Noreena L. Sweeney, Robert D. Bongard, Raulia Syrlybaeva, Ankan Gupta, Edgar Del Carpio, Marat R. Talipov, Costanza Garcia-Keller, Debbie C. Crans, Ramani Ramchandran, Daniel S. Sem

**Affiliations:** 1 Center for Structure-based Drug Design and Development, Department of Pharmaceutical Sciences, Concordia University Wisconsin, Mequon, WI, United States; 2 Department of Chemistry and Biochemistry, New Mexico State University, MSC 3C, Las Cruces, NM, United States; 3 Department of Pediatrics, Division of Neonatology, Children’s Research Institute (CRI) Developmental Vascular Biology Program, Translational and Biomedical Research Center, Milwaukee, WI, United States; 4 Department of Chemistry, Colorado State University, Fort Collins, CO, United States; 5 Department of Pharmacology and Toxicology, Medical College of Wisconsin, Milwaukee, WI, United States; 6 Cell and Molecular Biology Program, Colorado State University, Fort Collins, CO, United States

**Keywords:** DUSP5, pERK, phosphatase, regulation, enzyme kinetics, phosphorylated peptide substrates, dual active site, vanadate

## Abstract

**Introduction:**

Dual specific phosphatases (DUSPs) are mitogen-activated protein kinase (MAPK) regulators, which also serve as drug targets for treating various vascular diseases. Previously, we have presented mechanistic characterizations of DUSP5 and its interaction with pERK, proposing a dual active site.

**Methods:**

Herein, we characterize the interactions between the DUSP5 phosphatase domain and the pT-E-pY activation loop of ERK2, with specific active site assignments. We also report the full NMR chemical shift assignments of DUSP5 that now enable chemical shift perturbation and dynamics studies.

**Results and Discussion:**

Both phosphates of the pT-E-pY tripeptide are dephosphorylated, based on ^31^P NMR; but, steady state kinetic studies of the tripeptide both as a substrate and as an inhibitor indicate a preference for binding and dephosphorylation of the phospho-tyrosine before the phospho-threonine. Catalytic efficiency (k_cat_/K_m_) is 3.7 M^−1^S^−1^ for T-E-pY vs 1.3 M^−1^S^−1^ for pT-E-Y, although the diphosphorylated peptide (pT-E-pY) is a better substrate than both, with k_cat_/K_m_ = 18.2 M^−1^S^−1^. Steady state inhibition studies with the pNPP substrate yields K_is_ values for the peptide inhibitors of: 15.82 mM (pT-E-Y), 4.932 mM (T-E-pY), 1.672 mM (pT-E-pY). Steady state inhibition studies with pNPP substrate and with vanadate or phosphate inhibitors indicated competitive inhibition with Kis values of 0.0006122 mM (sodium vanadate) and 17.32 mM (sodium phosphate), similar to other Protein Tyrosine Phosphatases with an active site cysteine nucleophile that go through a five-coordinate high energy transition state or intermediate. Molecular dynamics (MD) studies confirm preferential binding of the diphosphorylated peptide, but with preference for binding the pY over the pT reside in the catalytic site proximal to the Cys263 nucleophile. Based on MD, the monophosphorylated peptide binds tighter if phosphorylated on the Tyr vs the Thr. And, if the starting pose of the docked diphosphorylated peptide has pT in the catalytic site, it will adjust to have the pY in the catalytic site, suggesting a dynamic shifting of the peptide orientation. 2D ^1^H-^15^N HSQC chemical shift perturbation studies confirm that DUSP5 with tripeptide bound is in a dynamic state, with extensive exchange broadening observed—especially of catalytic site residues. The availability of NMR chemical shift assignments enables additional future studies of DUSP5 binding to the ERK2 diphosphorylated activation loop.

**Summary:**

These studies indicate a preference for pY before pT binding, but with ability to bind and dephosphorylate both residues, and with a dynamic active site pocket that accommodates multiple tripeptide orientations.

## 1 Introduction

**FIGURE 1:**
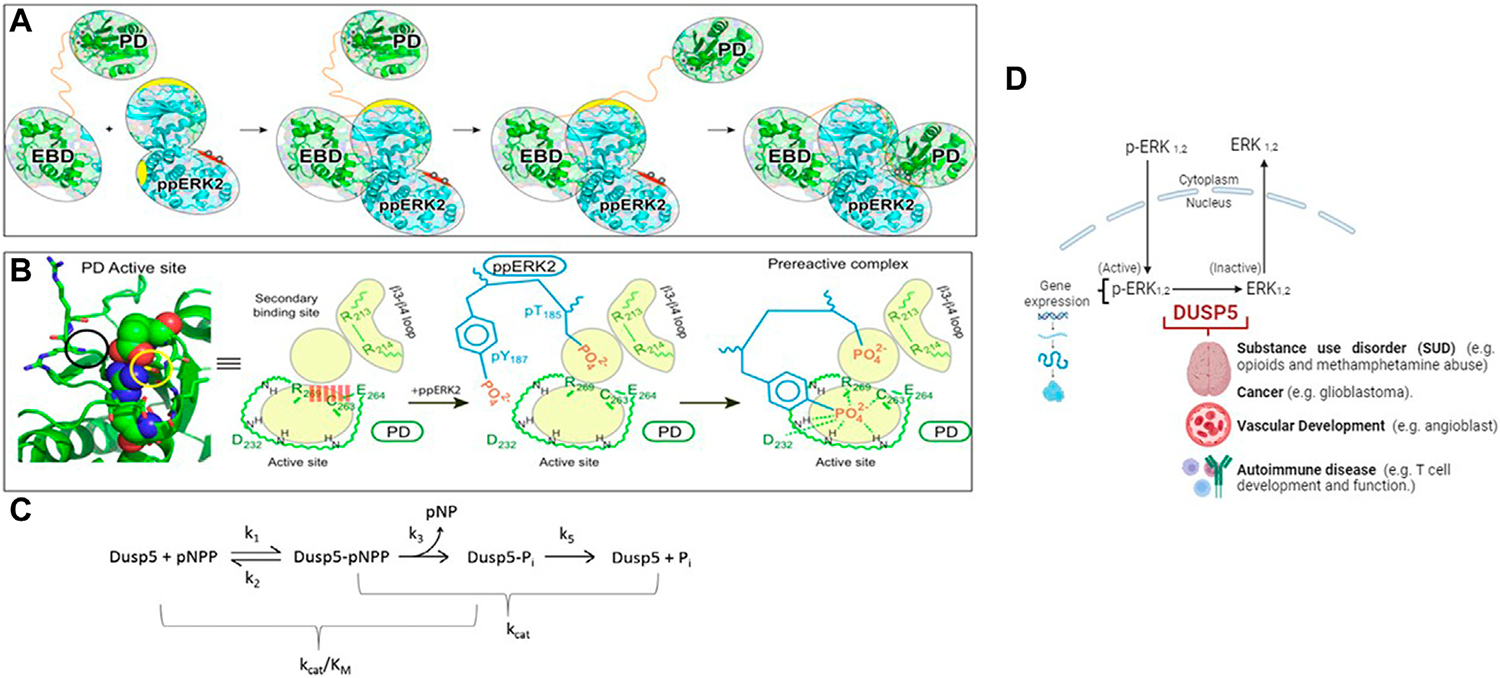
Model of the DUSP5-ERK2 mechanism. **(A, B)** Schematic representations of pERK binding to the DUSP5 phosphatase domain (PD) and ERK binding domain (EBD), leading to the near attack conformation. The corresponding kinetic scheme **(C)** shows that k_cat_/K_m_ reflects steps up to formation of the covalent adduct formed with the cysteine thiol nucleophile. Adapted from our prior studies (Talipov et al., 2016; Gupta et al., 2019). **(D)** Model illustrating the cellular-level impact of the DUSP5 - pERK interaction on biological function.

In eukaryotes, the prototypical mitogen-activated protein kinases (MAPK) have been widely studied and are comprised of a three-tier protein phosphorylation system (Cargnello and Roux, 2011; Raman et al., 2007). In this system, a series of proteins get phosphorylated in response to a ligand-receptor interaction and the signal leads into the nucleus to dictate gene expression. Extracellular regulated kinase (ERK), c-Jun N-terminal kinase (JNK) and p38 kinase are the terminal kinases in this prototypical pathway (Figure 1). As one would expect, exquisite control of the MAPK pathway is established by a second group of proteins called phosphatases (Caunt and Keyse, 2012). Dual specificity phosphatases (DUSPs) are a unique class of mitogen-activated kinase phosphatases (MKPs) that remove phosphates from both tyrosine and threonine residues of proteins (Caunt et al., 2008). The removal of phosphate groups renders the protein active or inactive based on the physiological status of the cell. Our laboratory studies the fifth member of DUSP family, DUSP5, which is responsible for dephosphorylating ERK in the nucleus (Kutty et al., 2017). ERK has two isoforms, ERK1 and ERK2, and they become fully active upon phosphorylation at two sites, namely, T^202/185^ & Y^204/187^ (Canagarajah, et al., 1997). Therefore, at any given time in a cell, there are four isoforms present, namely: nonphosphorylated ERK (np-ERK), monophosphorylated ERKs (pT-ERK or pY-ERK) or dual phosphorylated ERK (pTpY-ERK). ERKs are phosphorylated in the cytoplasm and the phosphorylated ERKs are transported into the nucleus where they exert their biological effect, such as gene expression. Maintaining pERK concentration in the nucleus is partly dictated by available pools of ERK isoforms for phosphorylation. Contribution to this ERK isoform pool can also arise from dephosphorylation of ERK. DUSP5 is responsible for dephosphorylation of both ERK1 and ERK2 in the nucleus (Kidger et al., 2017). DUSP5 removes phosphate groups from both T and Y residues, thereby inactivating ERK1/2. Therefore, the mechanism of dephosphorylation of one or both these ERK phosphates is important to understanding the cellular biology of ERK. Such a mechanistic understanding would also facilitate inhibitor development, with some inhibitors possibly having therapeutic applications.

Developments in the field of genetics and epigenetics have enabled the first wave of genome-wide analyses on people diagnosed with specific disorders. These analyses have provided critical insights, suggesting the involvement of distinct genes and biological processes in the onset of mental, neurological and vascular disorders. Increasing evidence has identified associations in the DUSP family with substance use disorder, cancer and vascular disorders (Ueda et al., 2003; Kuntz-Melcavage et al., 2009; Pramanik et al., 2009; Seleman et al., 2014; An et al., 2021). Our initial interest in investigating DUSP5:ERK2 dephosphorylation mechanism stemmed largely from the identification of a somatic mutation in DUSP5 (S147P) in patients diagnosed with vascular anomalies (Pramanik et al., 2009). The S147P mutation in DUSP5 causes hypoactivity of DUSP5 towards ERK2 (Nayak et al., 2014). To determine the molecular mechanism of DUSP5-mediated dephosphorylation of ERK2, we constructed a model of DUSP5/pT^185^pY^187^-ERK2 complex and noted in the X-ray structure of the DUSP5 phosphatase domain two phosphate binding sites, one in the active site where dephosphorylation occurs and the catalytic Cys263 resides, and one adjacent to the active site (Talipov et al., 2016). The presence of two phosphate pockets distinguishes DUSPs from other phosphatases which typically contain only the active site pocket. DUSP5 is also unique from other DUSPs in the presence of a catalytic Cys nucleophile.

Of mechanistic relevance for DUSP5, Protein Tyrosine Phosphatase 1B (PTP1B) also has a second site adjacent to the active site, which was found to be important in studies of vanadium-PTP1B complexes. The H-bonding to the five-coordinate protein adduct between the Cys thiol and the vanadium is stabilized by the many H-bonds to the vanadium and ligands in the first coordination sphere, as well at several H-bonds which extend into the adjacent site, further stabilizing the protein-inhibitor complex. The nature of the interplay between two adjacent sites in DUSP5 and PTP1B (Huyer et al., 1997; Zhang. and Zhang, 1998; Heo et al., 2002; Li et al., 2008; Feng et al., 2021) may provide a model for future DUSP5 inhibitor design, which has not yet been explored with all the phosphatases containing Cys in the active site (Lawrence and Van Etten, 1981; Zhou et al., 1993; Huyer et al., 1997; Zhang et al., 1997; Pannifer et al., 1998; Zhang. and Zhang, 1998; Reiter et al., 2001; Deng et al., 2002; Heo et al., 2002). This mechanism, where a Cys thiol attacks the phosphorylated peptide forming a protein-phosphate covalent adduct, was proposed for DUSP5 in our prior studies (Figure 1).

Our hypothesis in this work is that the two phosphate binding sites will be occupied by the phosphate groups (pT^185^& pY^187^) of ERK2. Indeed, molecular modeling studies confirmed this and further suggested the entry of the T185 residue present on the activation loop of ERK2 into the secondary DUSP5 binding site, which disrupts a salt bridge formed across the primary active site, thus facilitating entry of Y187 into the active site for subsequent phosphate hydrolysis (Figure 1). It is known that ERK2 is fully active when it is doubly phosphorylated (Canagarajah et al., 1997) and sequential dephosphorylation occurs with pY dephosphorylated first followed by pT. Whether the dephosphorylation events mediated by DUSP5 also follow a similar preferential sequence of events was not known. The studies performed herein partly address the mechanism and preference for the dephosphorylation reaction of ERK pT or pY residues in the DUSP5 pocket. The DUSP5-catalyzed ERK2 dephosphorylation mechanism, based on our prior studies, is summarized in Figure 1A–C (Talipov et al., 2016; Gupta et al., 2019). The biological role of DUSP5 is illustrated in Figure 1D, where it is shown that there are 2 isoforms of ERK (1 and 2); and, upon phosphorylation at two different sites (tyrosine Y187 and threonine T185), the ERKs become fully active. ERK is phosphorylated in the cytoplasm, crosses the nuclear membrane and exerts its biological effect via gene expression. DUSP5 is responsible for dephosphorylating ERK in the nucleus, controlling p-ERK activity by removing the phosphates from one or both sites. Dephosphorylated ERK 1 and 2 are inactive kinases. Therefore, it is critical to understand the mechanisms of phosphorylation and dephosphorylation of ERKs. Moreover, clinical research from genetic and epigenetics studies have identified associations between DUSP family genes and different mental, neurological and vascular disorders. Specifically, DUSP5’s role in substance use disorder, cancer, autoimmune diseases and vascular development. The mechanistic studies presented herein therefore provide a foundation for a better understanding of these downstream effects of ERK phosphorylation state.

## 2 Materials and methods

### 2.1 Protein expression and purification

The *Dusp5* phosphatase domain gene, both wild type (WT; DUSP5pdWT; used for kinetic studies) and the RDB version (DUSP5pdRDB; used for NMR studies), was synthesized by Blue Heron (Bothell, WA); and WT protein was expressed and purified as previously described (Nayak et al., 2014; Neumann et al., 2015). The DUSP5pdRDB construct used for the studies herein differs from the original wild type construct due to the addition of a thrombin cleavage site and the mutation of cysteine at position 263 to serine, to avoid catalytic turnover. This construct is identical to that which has been crystalized (2g6z), thereby permitting better comparison of NMR and x-ray data. The DUSP5pdRDB thrombin cleavage site also avoids additional N-terminal amino acids, which affected protein stability and quality of NMR spectra (Supplementary Figure S1).

Dual labeled (^13^C/^15^N) DUSP5pdRDB protein with an N-terminal hexa-histidine tag was expressed in BL21(DE3) cells grown in minimal media containing ammonium-^15^N chloride and ^13^C_6_ D-glucose, using a New Brunswick^™^ BioFlo^®^/Celligen^®^ 115 Fermentor/Bioreactor (Eppendorf). The vessel media temperature, dissolved oxygen, agitation rate and pH levels were maintained at 25°C, 100% room air oxygen, 400 rpm and pH 7.2, respectively. Protein expression was induced with 0.1 mM IPTG once the media O.D. at 600 nm reached 1.5. The cells were incubated an additional 14 h (h) and harvested by centrifugation. The resulting cell pellets were stored at −80°C. Dual labeled DUSP5pdRDB was isolated using a multi-step purification. The harvested cell pellets were lysed using sonication and the supernatant was passed through a Ni-sepharose Fast Flow resin column (GE Healthcare Life Sciences) to isolate the His-tagged protein. The histidine tag was cleaved with thrombin (1 unit thrombin per 100 μg protein), and a second Ni-sepharose column was used to isolate the protein without the histidine tag. Thrombin removal was accomplished by passage of the His-cleaved protein through a benzamidine column. The resulting sample was dialyzed (20 mM KH_2_PO_4_(pH 5.0), 75 mM KCl and 2 mM DTT) and concentrated to 1 mM by ultrafiltration in preparation for NMR spectroscopy analysis. Histidine tag cleavage and protein purification was monitored by SDS PAGE, with stacking and resolving gel compositions of 5% and 10%, respectively. See Supplementary Figure S2. Unlabeled protein was expressed and purified in a like manner, but using LB media rather than minimal media. NMR studies were performed using protein with a serine in the active site, whereas the wild type (WT) protein for enzyme kinetic studies had the cysteine (Cys263).

### 2.2 Multinuclear NMR studies of tripeptide reaction with DUSPpdWT

#### 2.2.1 Preparation of NMR samples and optimization of DUSP5RDB buffer conditions

Various NMR buffer conditions were explored, to determine conditions which gave maximum stability for NMR studies, and highest quality 2D HSQC spectra. The conditions examined were deuterated Tris buffer and potassium phosphate buffer, with pH values of 5.0 and 6.8. The procedure outlined below is for the 50 mM Tris, 100 mM KCl, and 2 mM DTT buffer samples at pH 5.0 and 6.8. This same procedure was followed for the investigation in buffer containing 20 mM potassium phosphate, 75 mM KCl, and 2 mM DTT.

An ^15^N labeled DUSP5pdRDB sample with a concentration of 500 μM was thawed on ice from −80°C storage. Samples without glycerol-d_8_ were prepared by transferring 300 μL of the ^15^N labeled protein and 30 μL D_2_O to a 1.5 mL Eppendorf tube. Glycerol-d_8_ containing samples were prepared in a similar fashion by transferring 300 μL of ^15^N labeled protein, 30 μL D_2_O, and 30 μL glycerol-d_8_ for a final concentration of 10% D_2_O and 10% glycerol-d_8_. The samples were spun down in a centrifuge at 14,000 rpm for 2 min to remove any particulate. The supernatant was transferred to a Shigemi NMR tube.

#### 2.2.2 ^31^P Multinuclear NMR studies of tripeptide reaction with DUSPpdWT

**FIGURE 2:**
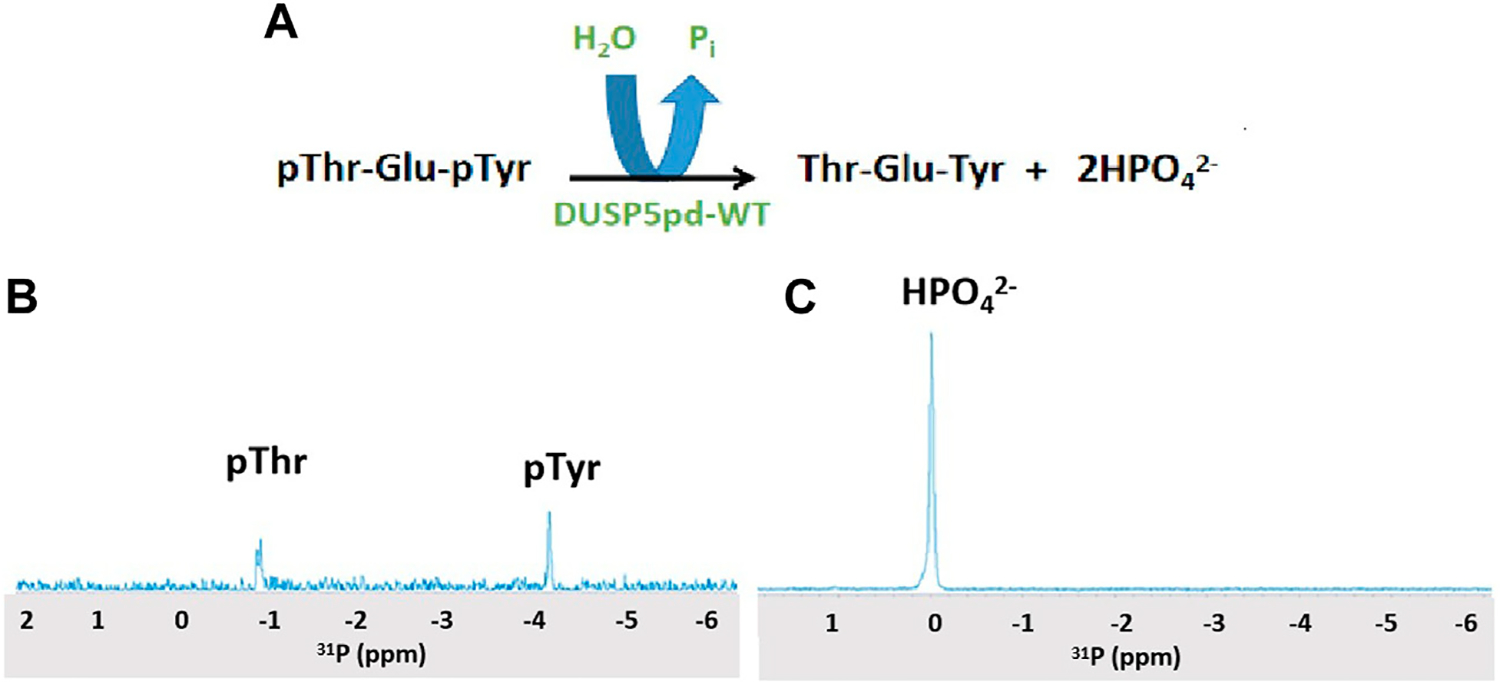
^31^P NMR spectra of the DUSP5-catalyzed hydrolysis of the pT-E-pY tripeptide. **(A)** The DUSP5-catalyzed hydrolysis reaction. **(B)** ^31^P NMR spectrum of 1 mM pT-E-pY tripeptide before adding enzyme, showing signals for phospho-threonine (doublet) and phospho-tyrosine (singlet). **(C)** ^31^P NMR spectrum 3 min after addition of 100 μM DUSP5pdWT, showing 100% conversion to orthophosphate. Reactions with lower enzyme concentration showed no clear preference for hydrolysis of pThr or pTyr (Supplementary Figure S3).

Hydrolysis of the pThr-Glu-pTyr peptide was monitored using ^31^P 1D NMR and a Varian 500 MHz spectrometer equipped with a broad band probe. 1 mM of peptide was incubated with 100 μM of DUSPpdWT protein for 3 min, and spectra were collected at 25°C. See Figure 2 and, for a time course, Supplementary Figure S3.

#### 2.2.3 2D ^1^H-^15^N HSQC spectra of DUSP5pdRDB protein labeled with ^15^N and ^13^C

2D ^1^H-^15^N HSQC spectra were collected using a 500 MHz Varian NMR spectrometer of a sample of expressed and purified DUSP5pdRDB protein labeled with ^15^N and ^13^C. The temperature for the experiments was adjusted to either 25°C, 30°C, or 37°C. After the sample was done running on the NMR spectrometer it was transferred to an oven set to either 25°C, 30°C, or 37°C. The samples were held at their respective temperatures for a total of 72 h 2D ^1^H-^15^N HSQC experiments were run on each sample after the completion of each 24 h period until 72 h. Final optimized conditions, based on quality of 2D ^1^H-^15^N HSQC spectra, were determined to be 20 mM potassium phosphate (pH 5), 75 mM KCl, 2 mM DTT with 10% glycerol, with NMR experiments run at 25°C. These are the conditions used for the 3D NMR experiments, and for chemical assignments, using the DUSP5pdRGB construct (Supplementary Figure S1).

#### 2.2.4 2D and 3D NMR and chemical shift assignments

Using these optimized conditions, protein chemical shift assignments were made for ^15^N and ^13^C labeled and cleaved DUSP5pdRDB protein (summarized in Supplementary Table S1). The multidimensional NMR experiments were performed on a 600 MHz Bruker NMR spectrometer equipped with a cryoprobe, using instrumentation at Medical College of Wisconsin.

The sample was concentrated to 1 mM in a total volume of 0.3 mL. 30μL glycerol-d_8_ was added to the sample and it was flash frozen and stored in the −80°C freezer until ready to analyze with the NMR spectrometer. 30μL D_2_O and 0.02% sodium azide were added to the sample. The sample was transferred to a Shigemi NMR tube and a standard suite of 2D and 3D experiments were collected for structure determination: 2D ^1^H-^15^N HSQC, 3D HNCO, HN(CO)CA, HN(CO)CACB, CCONH, HN(CA)CO, HNCA, and HNCACB.

Spectral data were processed using NMRpipe, then experiments were viewed in Xeasy and were peak picked. Once all the peaks were picked in all of the experiments, the data were run through Garant, which was able to auto assign roughly 85% of the chemical shifts. Next, the Garant assignments were opened into the HSQC experiment in Xeasy. The assignments were checked and corrected if necessary. This procedure was repeated for the HNCO experiment. The correct peak list of the HNCO experiment were loaded into the HN(CO)CA. The corrected HN(CO)CA list was loaded into the HNCACB, and the HN(CO) CACB experiments. Lastly, the HN(CO)CACB peak list was loaded into the CCONH experiment. The completed peak list was once again opened into the HSQC file for a final check of chemical shifts. These chemical shifts were saved to the prot file, with final assignments provided in Supplementary Table S1. Assignments mapped onto 2D ^1^H-^15^N HSQC spectra are in Supplementary Figure S4, S5.

#### 2.2.5 Secondary structure assignments

**FIGURE 3:**
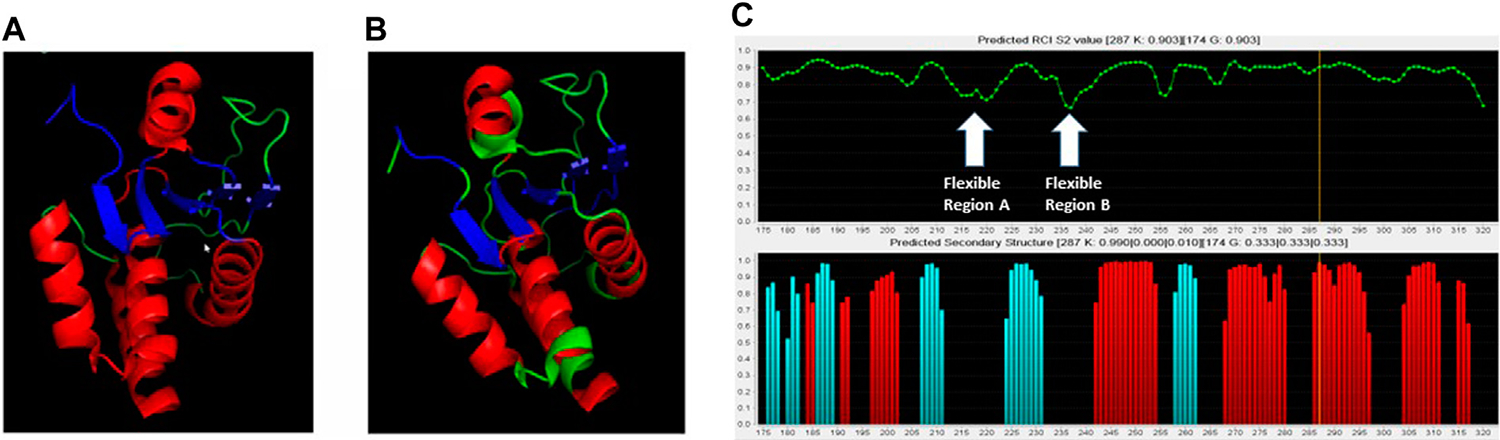
Comparison of crystal structure with NMR-based predictions. **(A)** Crystal structure of the DUSP5pd-WT protein (pdb code 2G6Z). **(B)** TALOS+ secondary structure predictions superimposed on the crystal structure. Red is for helix, Blue is for beta sheet and Green is for loop. **(C)** TALOS+ secondary structure and order parameter (flexibility) predictions. Two highly flexible regions **(A, B)** are identified, based on low predicted order parameter (S^2^) values.

The chemical shifts that were assigned for the primary structure were re-formatted for the TALOS+ web based secondary structure assignment (National Institute of Diabetes and Digestive and Kidney Diseases, 2014). Once the file was reformatted, the data file was uploaded to the TALOS+ server (Shen et al., 2009). This produced the analysis files. These files were then read through the jRAMA+ web-based java viewer for TALOS+ torsion angle predictions. These predictions were compared to the crystal structure, with pdb code 2G6Z (See Figure 3).

#### 2.2.6 DUSP5-tripeptide (pT-E-pY) NMR binding studies

**FIGURE 4:**
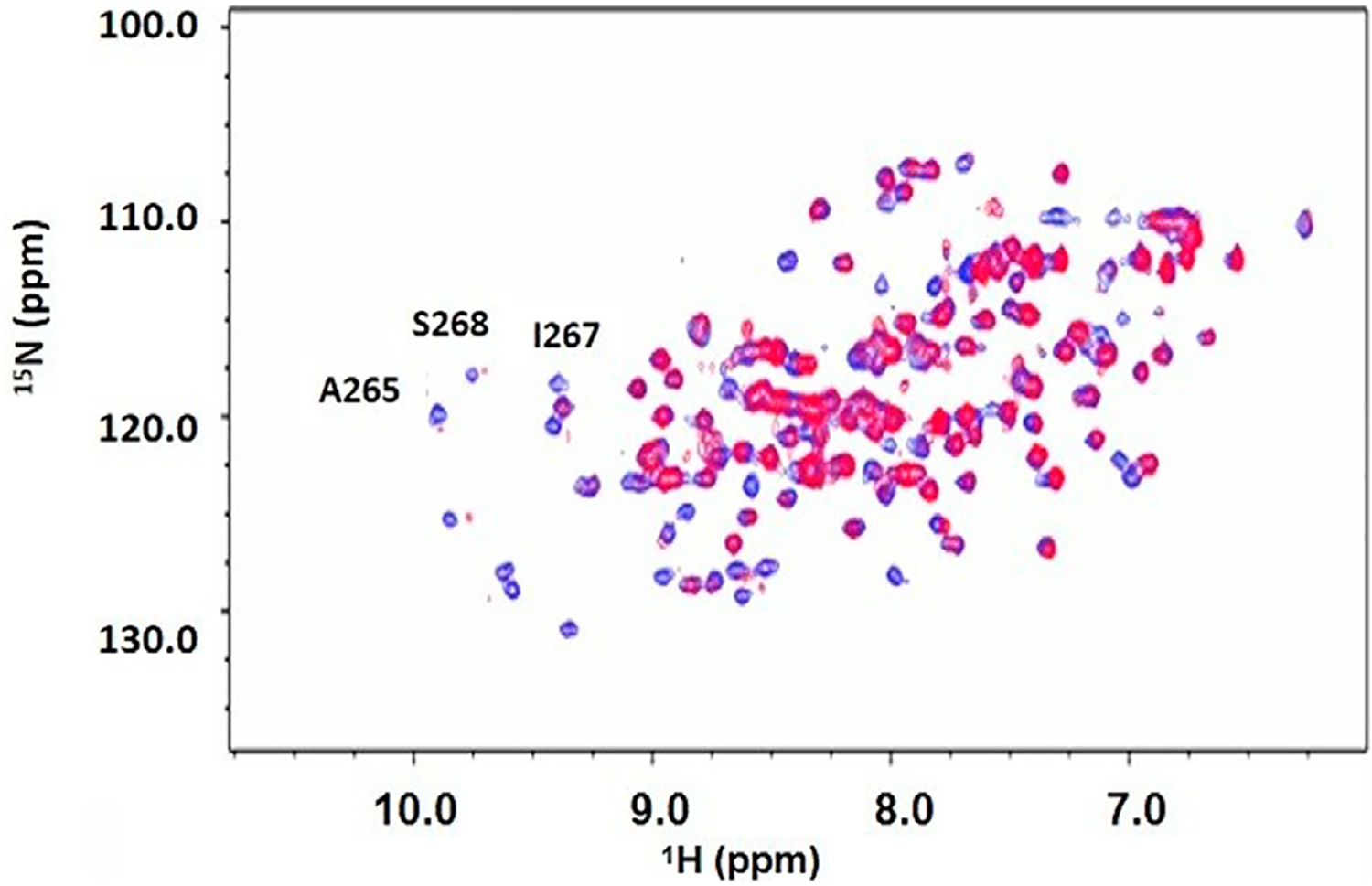
2D ^1^H-^15^N HSQC spectrum of DUSP5pdRDB ± tripeptide. ^1^H-^15^N HSQC spectrum for DUSP5pdWT protein alone (blue) or in the presence of the pT-E-pY tripeptide (red). Exchange broadening due to peptide binding occurred for many residues, including active site residues A265, S268 and I267. Full chemical shift assignments are provided in Supplementary Figure S4, S5, Supplementary Table S1).

**FIGURE 5:**
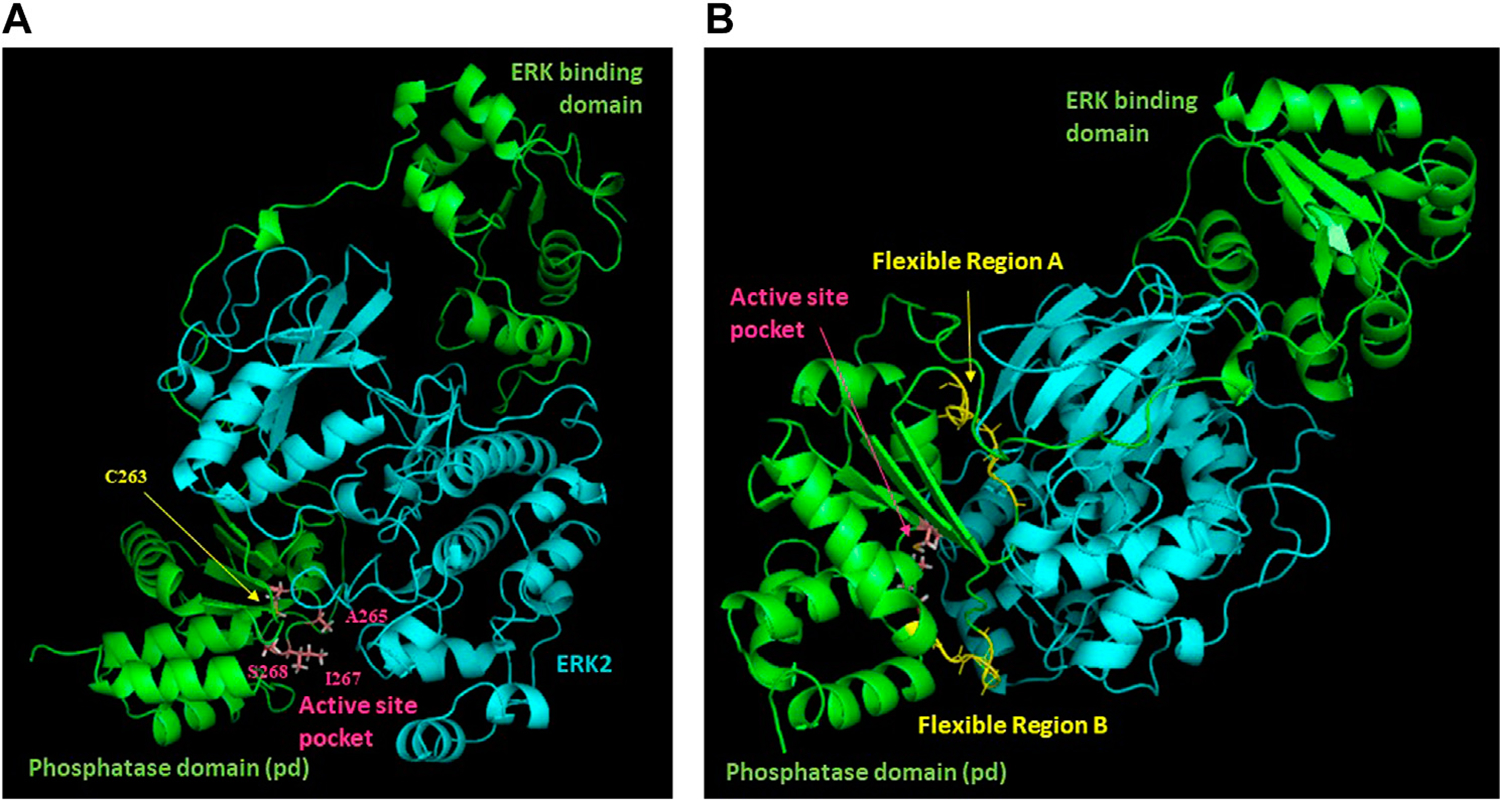
Model of the DUSP5-ERK2 complex. Results of NMR chemical shift analysis (Figures 3, 4) identifying exchange broadened active site residues **(A)** and dynamic regions with low predicted S^2^ values **(B)** have been mapped onto our previously reported model of the DUSP5-ERK2 complex.

After dialysis, two NMR sample tubes were prepared by adding 275 μL of 564 μM DUSP5pdRDB protein, 30 μL glycerol-d_8_, 30 μL D_2_O, and varying concentrations of the Ac-pThr-Glu-pTyr-NH_2_ (pT-E-pY) peptide (N-terminus acetylated and C-terminus amide) derived from pERK. Peptides were synthesized (Biomatik LLC, Wilmington, Delaware) to greater than 98% purity as determined by HPLC. The concentration of the tripeptide added was 0.250 mM. The Eppendorf tubes were centrifuged at 14,000 rpm for 2 min. After centrifugation the supernatant of each Eppendorf was transferred to each of two Shigemi NMR tubes. 2D ^1^H-^15^N HSQC spectra were collected on a 500 MHz Varian NMR spectrometer, at 25°C. Spectra were processed then overlaid using vNMRDraw software (shown in Figure 4). Exchange-broadened active site residues (identified in Figure 4) as well as residues with low predicted order parameters (low S^2^; from Figure 3) are indicated in our previously reported model of the DUSP5-ERK2 complex (Figure 5).

### 2.3 Steady state kinetic analysis of phosphate hydrolysis

#### 2.3.1 Steady state kinetic analysis of phosphate hydrolysis using a continuous Biomol Green assay

Peptide stock solutions were kept in buffer. Steady state kinetics were performed as described for the peptide inhibition studies, but monitoring actual dephosphorylation of the three tripeptides by DUSP5pdWT: pT-E-Y, T-E-pY, pT-E-pY. 1 mM of each tripeptide was incubated with 0.18 mM DUSP5pdWT enzyme at 25°C, and at timed intervals samples were analyzed for inorganic phosphate using the Biomol Green reagent (at 620 nm) (Supplementary Figure S6). Lack of protein aggregation at these concentrations is evidenced by consistently narrow linewidths in 2D HSQC spectra at various enzyme concentrations (i.e., minimal T2 broadening in Figs. 4, S4 and S5) as well as our previous data on dynamic light scattering of DUSP5 (Gupta et al., 2019). Kinetic studies were also repeated with varied tripeptide substrate concentration, to generate Michaelis-Menten curves. Results of fitting to Equation (1) are shown in Table 1.


(1) $$v=\frac{V_{\text{max}}\left[S\right]}{K_{m}+\left[S\right]}$$

#### 2.3.2 Steady state kinetic analysis of phosphate hydrolysis and inhibition by tripeptides

Recombinant DUSP5 PD (DUSP5pdWT) phosphatase activity was assayed as previously described, with minor modifications (Nayak et al., 2014; Neumann et al., 2015). All assays were performed in clear bottom 96-well microplates (Greiner 655001) with a total assay volume of 210 μL, using a SpectraMax M5 microplate reader (Molecular Devices). Assay buffer contained 100 mM Tris, 100 mM NaCl, 5 mM MgCl_2_·6H_2_O with 1 mM dithiothreitol (DTT) at pH 7.5. *p*-nitrophenol phosphate (*p*NPP, Sigma Aldrich) at 5 mM, a concentration near the established *K*_*m*_, was used for substrate. DUSP5 PD-mediated hydrolysis of *p*NPP to *p*-nitrophenolate, a chromogenic product which absorbs at 405 nm with a molar extinction coefficient of 18,000 M^−1^ cm^−1^, was monitored over time using the Spectramax M5 microplate reader. Wells that contained assay buffer with *p*NPP were used as blanks. Stock solutions of peptides were kept in buffer. Serial dilutions of each stock solution were prepared to generate a range of inhibitor concentrations for the steady state inhibition studies. Absorbance values were converted to *p*-nitrophenolate concentration using the product extinction coefficient and a micro well path length of 0.545 cm DUSP5 PD (DUSP5pdWT) enzyme progress curves over a range of substrate and peptide inhibitor concentrations were fitted globally using GraphPad Prism 6 to Equation (2) for steady-state competitive inhibition:

(2) $$v=\frac{V_{\text{max}}\left[S\right]}{K_{m}\left(1+\frac{\left[I\right]}{K_{i}}\right)+\left[S\right]}$$

where *v* is the initial velocity, *V*_*max*_ is the maximum velocity, *K*_*m*_ is the Michaelis constant, [*S*] is the *p*NPP concentration (varied from 1 to 80 mM), *I* is the peptide inhibitor concentration, and *K*_*i*_ the fitted inhibition constant. The parameters *V*_*max*_, *K*_*m*_, and *K*_*i*_ are shared in a global fit, resulting in one best-fit value for the complete data set (Table 2; Supplementary Figure S7; Supplementary Table S2).

#### 2.3.3 Steady state kinetic analysis of phosphate hydrolysis and inhibition by vanadate and phosphate

All assays were carried out using *p*NPP as a substrate in the presence of DTT using a microplate reader and a total volume of 100 μL. Each assay contained an enzyme concentration of approximately 0.072 mg/mL (appropriately 3.13 μM DUSP5pdWT) which was left in a stock solution at about 1 h at 4°C before use. Each assay consisted of a reaction mixture in 100 mM tris-hydrochloride buffer containing 100 mM NaCl and 5.0 mM magnesium chloride (MgCl_2_) at pH 7.5. The concentration of *p*NPP was varied from 2 to 25 mM (2, 4, 8, 15 and 25 mM) which was determined after measuring a K_m_ value of 8.02 mM using these conditions. The kinetic parameters were measured in the presence of 0.045 mM DTT in the assay solution and the assay was started with the addition of *p*NPP.

The inhibition by vanadate was carried out using inhibitor concentrations of 100, 200, 400, 600 and 800 nM under the assay conditions described above and in an assay solution containing 0.045 mM DTT. For phosphate inhibition studies, the phosphate inhibitor concentrations used were 0.5; 1; 2; 5; 10 mM. Both sets of inhibition studies are shown in Supplementary Figure S8, with both phosphate and vanadate behaving as competitive inhibitors. The kinetic runs were repeated three times and the data were initial processed using excel and the global kinetics analysis was performed using GraphPad Prism 10.1.2. The errors provided in the analysis were calculated by the program.

### 2.4 Docking and molecular dynamics

#### 2.4.1 Docking and molecular dynamics

Three series of molecular dynamics simulations with varied structural features of the molecules were conducted: neutral cysteine (Cys263) in the active site of DUSP5 and phosphorylated residues of the tripeptides in *dianionic* PO_3_^2−^ form (**Series 1**), deprotonated Cys263 and phosphorylated residues of the tripeptides in *dianionic* PO_3_^2−^ form (**Series 2**), deprotonated Cys263 and phosphorylated residues of the tripeptides in *monoanionic* PO_3_H^−^ form (**Series 3**). Results are summarized in Table 4 and Supplementary Figure S9, S10.

Calculations were done using methods described previously (Talipov et al., 2016; Gupta et al., 2019). Briefly, MD simulations of DUSP5pdWT were performed with the secondary binding site and active/catalytic binding sites loaded or unloaded with mono-phosphorylated ERK2 (i.e., pT-ERK2 or pY-ERK2) tripeptides described above (T-E-pY; pT-E-Y; also T-E-Y), while the remaining site was vacant and conformationally flexible in both cases. These simulations of the PD with the loaded secondary binding site or active site serve to probe the structural rearrangement of the active and secondary binding sites as compared with the original X-ray structure of PD domain. MD simulations were performed as before (Gupta et al., 2019) using the Amber MD software (Case et al., 2005) with the ff14SB force field with a 10.0 Å force cutoff and the Particle Mesh Ewald algorithm (Duan et al., 2003) to treat long-range electrostatic interactions. A triplicate 8 microsecond MD simulation (24 μs in total) was performed with each of the tripeptides: pT-E-pY, pT-E-Y, T-E-pY, and T-E-Y. Solvent was modeled explicitly with the TIP3P water model (Jorgensen et al., 1983). For each simulation, the initial structure (prepared as described below) was subjected to 500 steepest descent (SD) + 500 conjugate gradient (CG) minimization, in which protein structure was frozen, and subsequent 1000 SD + 1500 CG full minimization steps performed. The resulting structure was subjected to a warm-up NVT simulation with temperature being increased from 0 to 300K during 40 ps and subsequent 3-ns NPT equilibration. The sets of coordinates and velocities after each nanosecond of simulation were used as a starting point for three independent production run simulations. Production runs were performed using an NPT ensemble, with the time constant for heat bath coupling for the system equal to 10 ps and a Monte Carlo barostat. For each production run simulation, it was confirmed that temperature, mass density, and potential energy were nearly constant and did not show any noticeable drift.

**FIGURE 6:**
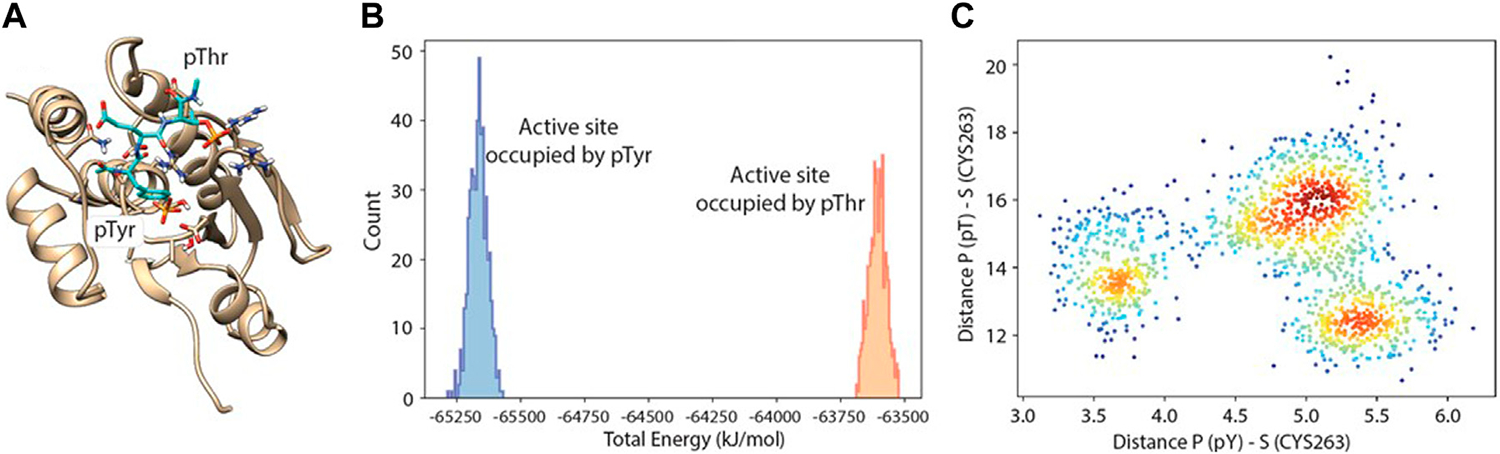
Molecular dynamics-docking population analysis. **(A)** Structural model of DUSP5 with the pT-E-pY (pThr-Glu-pTyr) peptide docked into the active site. **(B)** Relative population and total potential energy of binding modes with pY (blue) vs pT (gold) in the active site pocket, indicating that after MD, the preferred binding mode has pY in the active site pocket rather than pT. **(C)** A plot of distance from the active site serine to phosphate of pY vs phosphate of pT, indicating multiple low energy binding modes and preferential binding of pY to the active site pocket.

A docking study with molecular dynamics, as described above, was performed also using the fully phosphorylated peptide (pT-E-pY), and a population analysis was used to assess which of the two phosphate binding pockets was preferentially occupied by pT or pY, and at what energy (Figure 6).

The starting structure for simulations was the 2.7 Å resolution X-ray structure (PDB code 2G6Z) (Jeong et al., 2006), with Ser263 typically replaced with Cys, which is the WT (catalytically active) form. Structures were prepared using UCSF Chimera (Pettersen et al., 2004). Statistical analysis of the conformational dynamics was performed using R software version 3.3.1. For the cluster analysis, a hierarchical cluster analysis technique was used for the combined set of MD trajectories.

## 3 Results

### 3.1 Protein expression and purification for NMR studies

The DUSP5pdRDB construct (Supplementary Figure S1) was created with a thrombin cleavage site and the active site Cysteine 263 mutated to Serine to avoid catalytic turnover and oxidation during NMR experiments. Purification with a Nickel NTA column yielded pure protein for both steady state kinetic (DUSP5pdWT; with Cysteine 263) and NMR (DUSP5pdRDB; with Serine 263) studies (Supplementary Figure S2). The ^15^N-^13^C double labeled protein was well-behaved for >72 h, enabling collection of the suite of 3D NMR experiments needed to determine full chemical shift assignments (HNCO, HN(CO)CA, HN(CO)CACB, CCONH, HN(CA)CO, HNCA, and HNCACB); and, it gave well-dispersed ^1^H-^15^N HSQC spectra, indicative of a well-folded and stable protein (Supplementary Figure S4, S5). Chemical shift assignments were obtained and are summarized in Supplementary Table S1.

#### 3.1.1 ^31^P NMR-based characterization of phosphate hydrolysis of the pT-E-pY tripeptide

DUSP5 is a dual specificity phosphatase that cleaves both phospho-threonine (pT) and phospho-tyrosine (pY) residues, and it does this via a two-pocket gated mechanism that we have previously described (Figure 1). The peptide from the ERK activation loop that is dephosphorylated by DUSP5 actually contains proximal phosphates on both of these residues, separated by a glutamate: pT-E-pY. Studies presented herein characterize the binding and reaction of this tripeptide, derived from ERK. This pT-E-pY tripeptide is a substrate for DUSPpdWT with both phosphates cleaved 100%, based on ^31^P NMR analysis of the reaction progress (Figure 2). This result demonstrates that both the phosphate on the pY and the pT residues can be hydrolyzed by DUSPpdWT.

Hydrolysis was also examined at 10-fold lower enzyme concentration (Supplementary Figure S3) with the objective of observing a preference for either the pT or the pY residue. At the first timepoint after 3 min incubation, about 50% conversion was observed. Thus, about 25% each of the pT and pY peptides remained even after prolonged incubation. While both phosphates (pY and pT) are hydrolyzed efficiently in ^31^P NMR studies at 10-fold higher enzyme concentration, the fact that no more hydrolysis is occurring at lower enzyme concentration suggests either the enzyme is decomposed, or the enzyme is inhibited. In addition, the question remains as to whether there is differential affinity or binding order for pT or pY in each of the two binding pockets of DUSP5 (Figure 1), and how the enzyme catalyzed reaction occurs under steady state conditions.

#### 3.1.2 Protein NMR-based secondary structure prediction and pT-E-pY tripeptide binding study

Based on DUSP5 chemical shift values, secondary structure was predicted, with these predictions mapped onto the known DUSP5 x-ray crystal structure in Figure 3B. NMR data confirm that the solution structure is the same or at least consistent with the crystal structure, with perhaps more flexibility (loop-like structure) observed in some regions at the end of helices (Figure 3). Furthermore, the NMR data yielded a prediction of high flexibility (low S^2^ values) in regions 215–220 and 232–237, which we term “*Flexible Regions A and B*.” Extensive exchange broadening in 2D ^1^H-^15^N HSQC spectra (Figure 4) is consistent with protein conformational mobility in general. Mapping the Flexible Regions onto our modeled DUSP5-ERK complex reveals that these two loop regions define the binding interface with ERK (Figure 5), perhaps positioning the DUSP5 phosphatase domain relative to ERK so that the ERK activation loop (containing the pT-E-pY peptide) fits into the DUSP5 active site pocket for hydrolysis to occur.

Chemical shift perturbation studies, comparing 2D ^1^H-^15^N HSQC spectra before and after binding of the pT-E-pY tripeptide, indicate there is significant exchange broadening of cross peaks (Figure 4), including primary active site residues Ala265, Ile267 and Ser268. This suggests that the DUSP5-(pT-E-pY) complex is in a dynamic rather than rigid state, consistent with our previously published mechanism (Figure 1). These residues are shown on the DUSP5 phosphatase domain structure in Figure 6A and are close in space to the catalytic Cys163 residue in the primary active site pocket. Their flexibility on the NMR exchange timescale (typically msec) is consistent with motions and flexibility in the primary active site pocket that may be necessary to transfer pY or pT from binding in one pocket to the next.

#### 3.1.3 Steady state kinetic analysis of phosphate hydrolysis of phosphorylated tripeptides

Steady state kinetic studies were performed with the tripeptides acting as substrates (vs inhibitors), with progress curves and Michaelis-Menten plots shown in Supplementary Figure S6. Fitted Michaelis-Menten kinetic profiles yielded a relative activity as a substrate, expressed as k_cat_/K_m_, of: *p*T-E-*p*Y > T-E-*p*Y > *p*T-E-Y (Table 1). This clear trend is only with the kinetic parameter k_cat_/K_m_, the “catalytic efficiency,” that reflects all steps from (and including) initial binding up to the first irreversible step (Figure 1C), which is most likely the (initial) phosphate transfer that creates the phosphor-enzyme adduct (as also reported by Brandão, et al. (2010), for PTP1B). What is not clear from these results is whether the pT-E-pY peptide binds sequentially in one site then the next, and—even though there is higher overall affinity for pY over pT—what the preference is in each of the two binding pockets. In other words, the binding mechanism needs further exploration, which we performed below using kinetic studies with *p*NPP as substrate and phosphorylated peptides and with molecular modeling.

#### 3.1.4 Steady state kinetic analysis of *p*NPP hydrolysis inhibited by phosphorylated tripeptides

Steady state inhibition studies were performed with *p*NPP as substrate and the fully phosphorylated, nonphosphorylated, and both mono-phosphorylated T-E-Y tripeptides as inhibitors. The absorbances at 405 nm, measuring the dephosphorylation of pNPP, was analyzed with global fit analysis fitting to the equation for competitive inhibition (Supplementary Figure S7). The resulting kinetic parameters (K_m_, k_cat_, K_i_ and k_cat_/K_m_) summarized in Table 2 indicate an overall binding affinity preference and relative phosphor-tripeptide inhibition potency of: *p*T-E-*p*Y > T-E-*p*Y > *p*T-E-Y > T-E-Y. The diphosphorylated peptide is a roughly 3x more potent binder than the pY monophosphoryled peptide, which is in turn 3x more potent than the pT monophosphorylated peptide. But the greatest affinity boost comes from the addition of the initial phosphate group, since the pT monophosphoryleted peptide binds 30x stronger than nonphosphorylated peptide.

These data demonstrate that the phosphorylated peptides are inhibitory. While both pNPP and the phosphorylated peptide inhibitor can hydrolyze during the course of the assay, the rate of hydrolysis of pNPP is much faster than of the phosphorylated peptide. This can be rationalized because the pNPP concentration range is 2–20 mM whereas the peptides are present at much less than 1 mM, and the amount of the latter hydrolyzed is not contributing significantly to the overall level and rate of phosphate hydrolysis. Hence incubation of phosphorylated peptides with DUSP5pdWT will inhibit this enzyme and the observed ^31^P NMR results with low enzyme concentration where the reaction stops at 50% conversion can be explained at least in part by incubation of the phosphorylated peptide. Furthermore, these data show that the pY binds tighter than pT, consistent with the possibility that the initial binding of the diphosphorylated peptide will be through binding of pY. This hypothesis is explored further in molecule docking and dynamics experiments described below.

#### 3.1.5 Steady state kinetic analysis of pNPP hydrolysis inhibited by vanadate and phosphate

Since DUSP5 has an active site cysteine that is thought to act as a nucleophile (Figure 1), forming a Cys-phospho intermediate analogous to PTP1B, it might be expected DUSP5 is potently inhibited by vanadate, since Brandão, et al. (2009 and Brandao et al., 2010) had previously reported such a potent inhibitory adduct formed between vanadate and the cysteine thiol of PTP1B. Vanadate readily forms trigonal bipyramidal structures and are widely accepted as mimicking transition state or high energy intermediate structures for phosphate ester hydrolysis (Crans et al., 2014; Crans, 2015; McLauchlan et al., 2015). The structural advantage that vanadate has over other analogs including tungstate (WO_4_^2−^), molybdate (MoO_4_^2−^), magnesium fluoride (MgF_3_^−^), and nitrate (NO_3_^−^) is that it has the same overall charge as phosphate. Herein we explored the inhibition by vanadate and compared it with inhibition of phosphate, as our lab has done previously (Crans, 2015; McLauchlan et al., 2015). However, because of the redox properties of vanadate (Crans et al., 2004), it can be challenging to use in the presence of a reducing agent (DTT) that is needed to maintain the stability of protein phosphatase, with a readily oxidized cysteine in the active site (Crans et al., 2014; Crans, 2015; McLauchlan et al., 2015). We carried out preliminary studies with DUSP5 using varying concentrations of DTT. Tris forms a weak complex with vanadate, and therefore was compared to Hepes, which is a buffer with similar pK_a_ value that interacts minimally with vanadate (Crans et al., 1989). Preliminary studies in both buffers showed that some variations in K_m_ values for *p*NPP and K_i_ values for vanadate were observed. These preliminary studies allowed us to choose Tris buffer with minimum DTT concentration for studies with the DUSP5pdWT enzyme for comparison with the kinetic analysis carried out in this work.

Using the conditions determined by the *p*NPP K_m_ of 8.02 mM that was measured and calculated using global kinetics analysis, with vanadate at concentrations at 100, 200, 400, 600 and 800 nM, a K_i_ value of 638 nM was obtained for vanadate (Supplementary Figure S8). Considering that the active site of DUSP5 contains a cysteine residue and it has a secondary binding site which does not, DUSP5 is distinct from than other cysteine containing phosphatases. However, as shown by our data, we find that the inhibition is competitive and the K_i_ value is very potent, submicromolar. A summary of K_i_ values surveyed from the literature is listed in Table 3, and demonstrates the variations in K_i_ values reported for vanadate, and for comparison phosphate, considering the differences in phosphatases with cysteine in the active site, substrate, and pH values in the assay (Lawrence and Van Etten, 1981; Zhou et al., 1993; Huyer et al., 1997; Zhang et al., 1997; Zhang. and Zhang, 1998; Deng et al., 2002; Heo et al., 2002; Lu et al., 2012). Except for one bovine liver enzyme (Lawrence and Van Etten, 1981) these phosphatases all have micromolar or submicromolar K_i_ values. The most well studied vanadate-phosphatase system with a cysteine residue in the active site is protein phosphatase 1B (PTP1B) (Brandão et al., 2009; Brandão, et al., 2010; Crans, 2015). There are multiple X-ray structures of the vanadate-PTP1B complex showing a range of different complexes. One complex in particular has part of the peptide tyrosine-phosphate substrate bound to the PTP1B. The substrate is a hexapeptide with a sequence DADEYL which is a fragment of the epidermal growth factor receptor, which, in phosphorylated form (DADEpYL) exhibits strong affinity for PTP1B (Pedersen et al., 2004). A previous crystallographic study shows a Michaelis complex between a catalytically inactive C215S mutant of PTP1B and the phosphorylated peptide (Jia et al., 1995) but the second transition state of this protein with vanadate (Pedersen et al., 2004; Brandão, et al., 2010) has lost the tyrosine peptide. The crystal structure of the C215S mutant of PTP1B and the phosphorylated peptide shows the peptide extending from the active site to the adjacent site which provides a number additional stabilizing H-bonds and as such a system to which there is more direct comparison to DUSP5 than the other Cys-containing phosphatases.

For comparison, the inhibitory effect of phosphate on DUSP5 was also determined (Supplementary Figure S8B). Phosphate also exhibits a competitive inhibition pattern with DUSP5 and yields a K_i_ of 17.3 mM, similar to those reported previously (Lawrence and Van Etten, 1981; Zhang et al., 1997; Zhang. and Zhang, 1998). The considerably lower inhibitory effects of phosphate (5 orders of magnitude higher K_i_) compared to vanadate (K_i_ = 638 nM) is due to the fact that phosphate is not able to form the stable pentacoordinate complex in the trigonal bipyramidal structure that vanadate can adopt, resembling the transition state or high energy intermediate as reported for other protein phosphatases (McLauchlan et al., 2015). Our studies demonstrate that DUSP5 is subjected to the same effects during its catalytic cycle, and in this regard resemble PTP1B.

### 3.2 Molecular docking and dynamics of tripeptide binding

#### 3.2.1 Molecular docking and dynamics of tripeptide binding

Binding of the pT-E-pY tripeptide, and its monophosphorylated analogs, was explored using docking and molecular dynamics to assess preference for the two DUSP5 phosphate binding pockets (catalytic and the secondary site; Figure 1) and to verify if the pY residue binds tighter than the pT residue. A 24-microsecond molecular dynamics (MD) simulation of the DUSP5 complex with the pT-E-pY tripeptide was performed, and which showed that (a) the complex is stable and maintains pY in the catalytic site and pT in the secondary site (Figure 6A), (b) there is preferential binding of the pY group of pT-E-pY before pT (Figure 6B), (c) distance between the active site residue (Cys263) is shorter to pY than to pT (Figure 6C), and (d) the pY and pT groups bind in the catalytic (active) and the secondary binding site, respectively, with binding of pT in the secondary pocket permitting binding of pY in the active site, consistent with our published mechanism (Figure 1; Talipov et al., 2016). The results from the accelerated sampling of free energy profile for the complex formation (replica exchange umbrella sampling technique) are also consistent with the catalytic efficiency measurements: pT-E-pY (Δ*G* = −6.8 kcal/mol) > T-E-pY (−6.5 kcal/mol) > pT-E-Y (−6.3 kcal/mol) > T-E-Y (−5.9 kcal/mol).

Our calculations show that the active site (containing the catalytic residue Cys263) is preferred over the secondary active site for binding phosphorylated residues. Tripeptides pT-E-Y and T-E-pY bind to the active site (~5 Å) by their single phosphate groups in more than 50% of frames in all series in the MD simulation. pY is more likely to be involved in this interaction than pT. Numbers of frames with corresponding interactions for T-E-pY and pT-E-Y are about 80–90% and 50–60% respectively. Among all the series of simulations, the strongest interactions are observed in **Series 3** with deprotonated Cys263 and the phosphate groups in monoanionic PO_3_H^−^ form; and, **Series 2** with deprotonated Cys263 and phosphate groups in dianionic PO_3_^2−^ form showed poorest binding between modeled fragments, due to high negative charge of the ligands (Table 4; Supplementary Figure S9, S10).

In the case of a tripeptide with two phosphate groups (pT-E-pY), the main active site is occupied by the pY residue most of the time. The most illustrious results in this connection are from **Series 3**: the distribution between frames with Cys-pY and Cys-pT interactions are about 90% and 15% respectively (in 5% of cases both phosphorylated groups are located near Cys). According to the Boltzmann distribution, this ratio between conformers corresponds to 1.3 kcal/mol of difference in free energy. Calculations from **Series 2** show that two dianions make the ligand too negative for interaction with Cys^−^. In this case the active site in most of the frames is empty, while the occupation of the secondary binding site is considerably higher than in other sets of calculations. In simulations involving neutral cysteine, pT did not show interactions with the main active site, while pY is bound proximal to Cys in 40% of frames.

Since the single phosphate groups of pT-E-Y and T-E-pY tripeptides are tending to occupy the main active site, the corresponding secondary binding site is mostly empty, with the average distance between a center of mass of arginines ARG213–214 forming the second active site and phosphorylated residues of the tripeptides being about 10 Å (Supplementary Figure S10). The other reason for these long distances is that in most of the frames these arginines are not close to each other and do not form a tight active site. Residues interacting with only one arginine are located some distance from the center of mass.

## 4 Conclusion

In summary, based on NMR binding studies and the observation of competitive inhibition, the ERK tripeptides–in all phosphorylation states—appear to bind preferentially in the expected primary active site pocket, comprised of residues that include Cys263 (nucleophilic catalyst), Ser268, A265 and I267 (Figure 5). But the observation of significant NMR exchange broadening (Figure 4) suggests the DUSP5 protein undergoes dynamic motions on an intermediate timescale (probably msec), likely due to local conformational changes indicative of a somewhat dynamic protein capable of induced fit binding. This mobility is especially pronounced in loop regions we have defined as Flexible Regions A and B (Figure 3C). Such flexibility may be needed to accommodate binding of the ERK activation loop tripeptide in one of two potential catalytically competent orientations, with pY or pT in the active site pocket (with pY being favored). Surface loops on the DUSP5 phosphatase domain that interface with ERK2 appear especially flexible, based on low predicted S^2^ values, consistent with a flexible ERK-DUSP5 binding interface, to go along with a malleable DUSP5 active site.

The inhibition studies with vanadate demonstrate that the transition states and catalytic cycle of DUSP5 is likely to be affected similarly to that of other Cys-containing phosphatases, such as PTP1B. We therefore explored the interaction of vanadate with DUSP5 and compared with phosphate. Although DUSP5 is unique in that it has two phosphate binding pockets, an active site and a secondary binding pocket, one for the phosphorylated tyrosine and one for the phosphorylated threonine. These sites likely flex upon peptide binding, with associated conformational changes. The crystal structure of the phosphotyrosine complex of PTP1B has provided a snapshot of its catalytic cycle; but, that phosphatase has only one active site. DUSP5’s substrate extends into an adjacent binding pocket. These interesting similarities with PTP1B will be explored further in future work.

Based on docking and molecular dynamics (MD) studies, the phosphorylated residues (pY; pT) of the ERK activation loop tripeptides can readily bind to the active site of DUSP5pdWT, with a preference for the pY residue binding in the active site pocket relative to the secondary binding site. Still, the MD simulations indicate that both binding orientations are possible, even if pY in the main active site pocket is preferred. This preference for pY is consistent with the higher affinity for the T-E-pY peptide relative to the pT-E-Y peptide in steady state kinetic inhibition studies; and, with the observation that the T-E-pY peptide is more readily hydrolyzed based on the steady state k_cat_/K_m_ values. Thus, pY binding in the active site pocket, proximal to the catalytic Cys163 residue, is more favorable. But, pT can occupy this site as well and is also readily hydrolyzed, with only 3-fold lower k_cat_/K_m_ relative to pY. Importantly, DUSP5 is behaving as other Cys-containing protein phosphatases, which are all potently inhibited by vanadate, due to vanadate’s ability to form a stable five-coordinate transition state protein complex.

Future studies will be directed towards further characterizing the structural and dynamics changes associated with DUSP5 binding to ERK and ERK-derived phosphorylated peptides, enabled by the presentation herein of full NMR chemical shift assignments. These studies will enable the design of inhibitors as mechanistic probes, and potential drug leads. Potential candidate inhibitors could include transition state analogs such as vanadate or derivatives obtained from information gained in studies using a vanadate-containing potent transition state analog.

The literature has shown the critical role that DUSP5 plays in disorders that include vascular disease, cancer, autoimmune disease and substance use disorder (Figure 1D). In particular, increases or decreases in DUSP5 mRNA expression were observed in different brain structures involved in reward circuits like the prefrontal cortex and nucleus accumbens, respectively. Moreover, studies suggest that DUSP5 has a role in drug exposure as well as synaptic plasticity during drug-seeking behavior (relapse) (Kuntz-Melcavage et al., 2009; Seleman et al., 2014; An et al., 2021). Future studies in our laboratories will be directed to understanding these biological processes underlying or mediated by DUSP5, and their biological relevance in the context of substance use disorder.

## Supplementary Material

Supplementary Figures

## Figures and Tables

**TABLE 1:** DUSP5pdWT kinetic parameters (±SE) estimated from Michaelis-Menten fits (Eq. 1) of substrate velocity curves with the indicated tripeptides as *substrates*. Velocities were determined from the rates of inorganic phosphate generation, monitored with Biomol green reagent.

Peptide	k_cat_ (S^−1^)	K_m_ (mM)	k_cat_/K_m_ (M^−1^s^−1^)
pT-E-pY	7.80 ± 0.62 × 10^−2^	4.3 ± 0.6	18.2
T-E-pY	4.56 ± 0.63 × 10^−2^	12.2 ± 1.5	3.7
pT-E-Y	1.67 ± 0.45 × 10^−2^	12.8 ± 5.2	1.3

**TABLE 2:** Kinetic parameters for inhibition of DUSP5pdWT hydrolysis of para-nitrophenol phosphate (*pNPP*) in the presence of pERK activation loop tripeptide mimetics as *inhibitors*, having different phosphorylation states. Kinetic parameters estimated from global non-linear regression competitive inhibition model fits (Michaelis-Menten) to Eq. 2.

Global Competitive Enzyme Inhibition Model: Michaelis-Menten Fits				
Kinetic Parameters	Peptides			
	pT-E-pY	T-E-pY	pT-E-Y	T-E-Y
k_cat_ (s^−1^)	2.03 ± 0.07 × 10^−2^	1.82 ± 0.04 × 10^−2^	1.71 ± 0.04 × 10^−2^	1.92 ± 0.05 × 10^−2^
K_m_ (mM)	10.8 ± 1.2	8.56 ± 0.68	6.50 ± 0.68	8.41 ± 0.90
K_i_ (mM)	1.67 ± 0.20	4.93 ± 0.55	15.8 ± 5.2	583 ± 764
k_cat_/K_m_ (M^−1^s^−1^)	1.88	2.13	2.63	2.28

**TABLE 3:** K_i_ values for vanadate and phosphate measured in various phosphatases.

Enzyme source	Enzyme details	Active site residue	pH of assay	Ki (M)	Substrate	Assay buffer	References
**Vanadate as inhibitor**							
Bovine heart	Bovine Low Molecular Weight Phosphotyrosyl Phosphatase	S/Cys	5.0 7.5	5.4 ± 0.8 × 10^−6^ 1.0 ± 0.6 × 10^−6^	PNPP	Sodium acetate	Zhang et al. (1997)
Bovine liver	Acid phosphatases	S/Cys	5.5	2 × 10^−3^	PNPP	Sodium acetate	Lawrence and Van Etten (1981)
Human	PTP1B	S/Cys	7.3	3.8 ± 0.2 × 10^−7^	FDP	HEPES	Huyer et al. (1997)
Human	Human prostatic acid phosphatase	S/Cys	5.0	3.6 × 10^−5^	PNPP	Sodium acetate	Zhou et al. (1993)
Human	Phosphotyrosyl protein phosphatases	S/Cys	5.0	2.4 × 10^−5^	PNPP	Sodium acetate	Zhou et al. (1993)
Human	PTP1B	S/Cys	7.5	5.6 ± 0.8 × 10^−6^	PNPP	Tris	Heo et al. (2002)
Human	PTP1B	S/Cys	5.5	4.1 ± 0.8 × 10^−4^	PNPP	Tris	Heo et al. (2002)
Human	SHP-1 (domain containing protein tyrosine phosphatase-1)	DNM	7.5	9.3 ± 0.9 × 10^−6^	PNPP	Tris	Heo et al. (2002)
Yersinia	PTPase	S/Cys	5.5	1.1 × 10^−6^	PNPP	Acetate	Deng et al. (2002)
Yersinia	PTPase	S/Cys	7.5	1.2 × 10^−6^	PNPP	Tris	Deng et al. (2002)
Lambda phage	Bacteriophage λ protein phosphatase	DNM	7.8	7.0 ± 2.0 × 10^−7^	PNPP	Tris	Reiter et al. (2001)
**Phosphate as inhibitor**							
Bovine heart	Bovine Low Molecular Weight Phosphotyrosyl Phosphatase	S/Cys	5.0 7.5	2.2 ± 0.4 × 10^−3^ 6.2 ± 1.0 × 10^−3^	PNPP	Sodium acetate	Zhang et al. (1997)
Bovine liver	Acid phosphatases	S/Cys	5.5	3 × 10^−3^	PNPP	Sodium acetate	Lawrence and Van Etten (1981)
Human	PTP1B	S/Cys	7.0	1.7 × 10^−2^	PNPP	3,3-Dimethylglutarate	Zhang, Y.L. and Zhang, Z.Y. (1998)

**TABLE 4:** Molecular Dynamics Series for pERK activation loop tripeptide tripeptides bound to DUSP5.

	Starting structure		MD simulation	
Series 1: CYS263(SH) and (T/Y)-OPO_3_^2−^				
Phosphorylation State	Main	Secondary	Main	Secondary
pT-E-pY	pT	pY	-	pT (2–3 Arg)
	pY	pT	pY	pT (1 Arg)
pT-E-Y	pT	-	pT	-
T-E-pY	pY	-	pY	-
Series 2: CYS263(S^−^) and (T/Y)-OPO_3_^2−^				
Phosphorylation State	Main	Secondary	Main	Secondary
pT-E-pY	pT	pY	-	pT (2–3 Arg)
	pY	pT	-	pT (2–3 Arg)
pT-E-Y	pT	-	pT	-
T-E-pY	pY	-	pY	-
Series 3: CYS263(S^−^) and (T/Y)-OPO_3_H^−^				
Phosphorylation State	Main	Secondary	Main	Secondary
pT-E-pY	pT	pY	pY	pT (1Arg)
	pY	pT	pY	pT (1Arg)
pT-E-Y	pT	-	pT	-
T-E-pY	pY	-	pY	-

## Data Availability

The original contributions presented in the study are included in the article/Supplementary Material, further inquiries can be directed to the corresponding authors.
