# Supplementary material for: Structural and kinetic characterization of DUSP5 with a Di-phosphorylated tripeptide substrate from the ERK activation loop
Source: Front Chem Biol. Author manuscript; Available in PMC 2025 Jan 2. (PMC11694514; doi:10.3389/fchbi.2024.1385560)
Supplement: Supplementary Figures [file NIHMS2042009-supplement-Supplementary_Figures.pdf]

Supplementary Figures

1. His tag
2. TEV recognition sequence
3. Sgf1 5' restriction site
4. Thrombin recognition sequence (cleaves between arginine and glycine)

His tag cleavage should decrease the molecular weight from 19,326 to 16,799

1. 2. 3. 4.

(MHHHHHHENLYFQGAIALVPR)GSHMGPVEILPFLYLGSAYHASKCEFLANLHITALLNVSRRTSEACMTH
LHYKWIPVEDSHTADISSHFQEAIDFIDCVREKGGKVLVHSEAGISRSPTICMAYLMKTKQFRLKEAFDYIKQ
RRSMVSPNFGFMGQLLQYESEILPST∗***stop codon***

DUSP5pdRDB sequence with His_6 tag cleaved:

(2G6Z)

GSHMGPVEILPFLYLGSAYHASKCEFLANLHITALLNVSRRTSEACMTHLHYKWIPVEDSHTADISSHFQEAI
DFIDCVREKGGKVLVHSEAGISRSPTICMAYLMKTKQFRLKEAFDYIKQRRSMVSPNFGFMGQLLQYESEIL
PST

Fig. S1. DUSP5pdRDB sequence for expressed protein (phosphatase domain only), before and after Thrombin cleavage. The active site nucleophile is Cysteine-263 (DUSP5pdWT construct), which is shown here mutated to Serine (DUSP5pdRDB construct), in red. The catalytically inactive DUSP5pdRDB construct was used for NMR structural analysis, and the DUSP5pdWT construct was used for all kinetic studies.

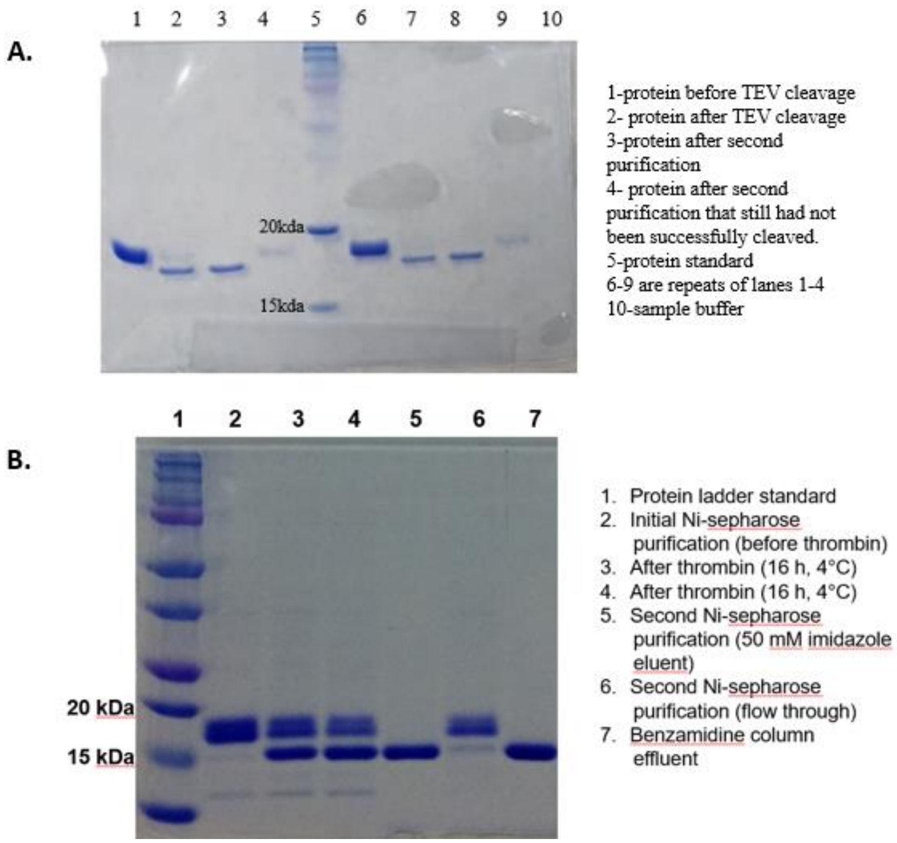

Fig. S2. (A) SDS PAGE gels for various steps in the DUSP5pdWT expression in *E. coli* and purification. (B) Gel analysis for various steps in the purification of double (^{13}C-^{15}N) labeled DUSP5pdRDB protein, used for NMR studies.

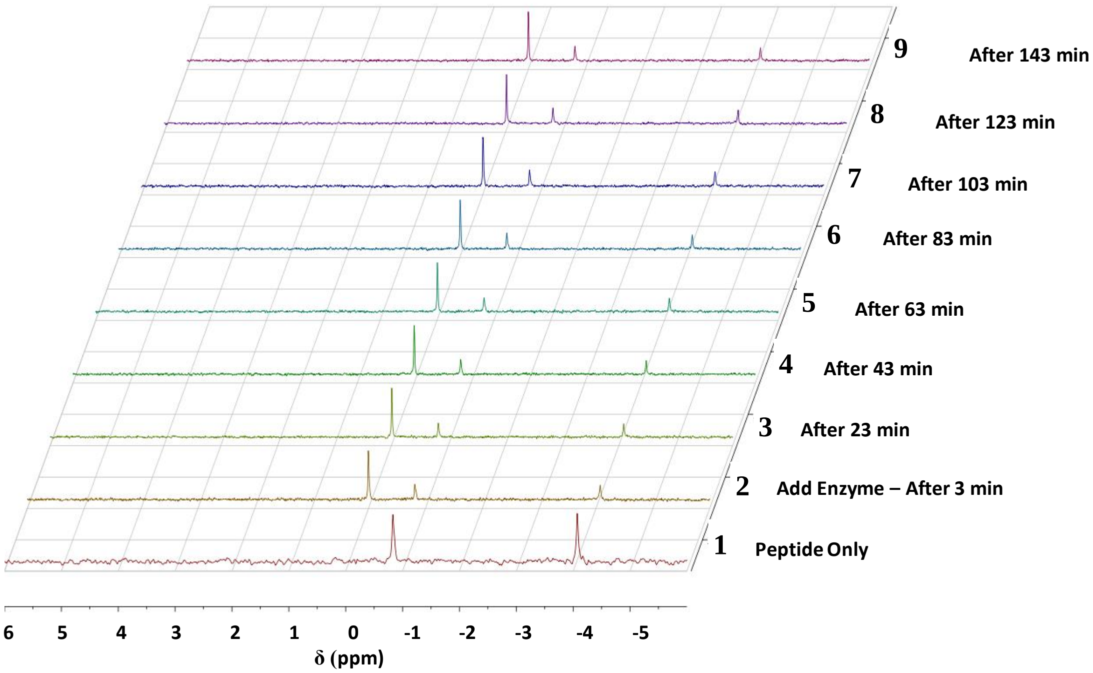

Fig. S3. ^{31}P NMR spectra of the DUSP5-catalyzed hydrolysis of the pThr-Glu-pTyr tripeptide. ^{31}P NMR spectra of 1 mM tripeptide before and after adding 10 mM DUSP5pdWT. Reactions showed no clear preference for hydrolysis of pThr or pTyr, and appeared to stop before reaching completion.

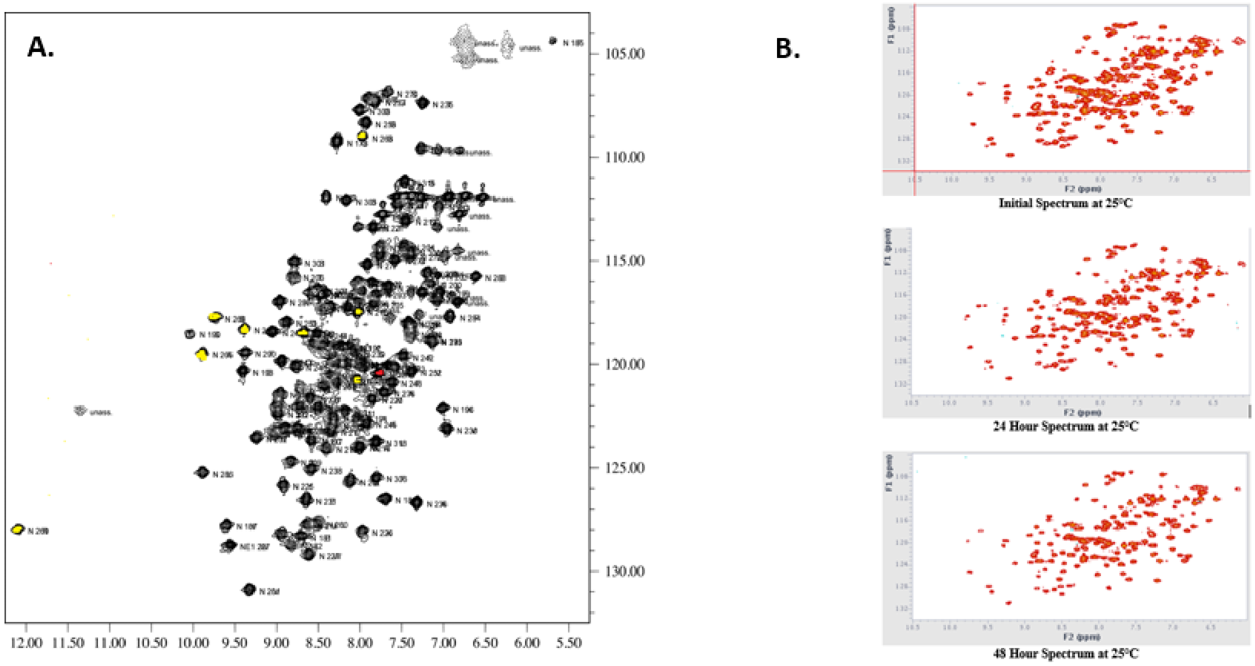

Fig. S4. 2D ^{1}H-^{15}N HSQC Spectrum of DUSP5pdRDB. (A) ^{1}H-^{15}N HSQC spectrum with chemical shift assignments, based on 3D NMR spectra: HNCO, HN(CO)CA, HN(CO)CACB, C(CO)NH, HN(CA)CO, HNCA and HNCACB. Cross peaks for assigned active site residues, based on the crystal structure, have been identified. Full chemical shift assignments are in Supplementary Table S1. **(B)** Change in the observed ^{1}H-^{15}N HSQC spectrum over time. Intensity of peaks is decreasing as protein precipitates, but no significant change in linewidth is observed (i.e. no apparent change in T2 do to protein aggregation state).

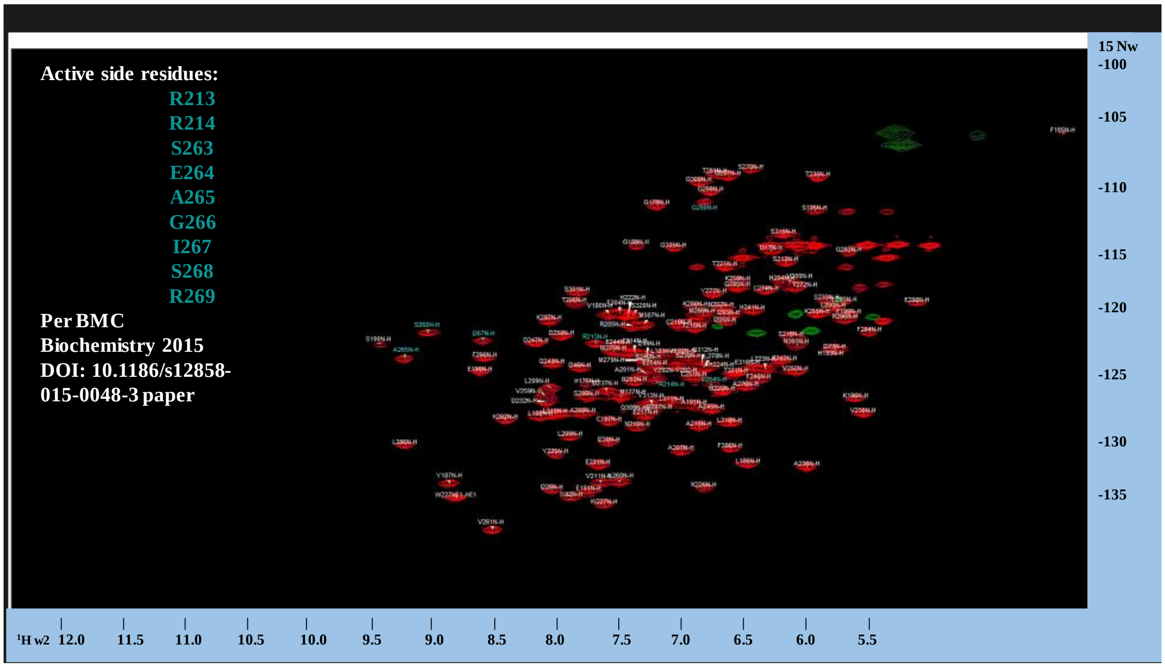

Fig. S5. 2D ^{1}H-^{15}N HSQC Spectrum of DUSP5pdRDB. ^{1}H-^{15}N HSQC spectrum with chemical shift assignments, based on 3D NMR spectra. Cross peaks for assigned active site residues, based on the crystal structure, have been identified.

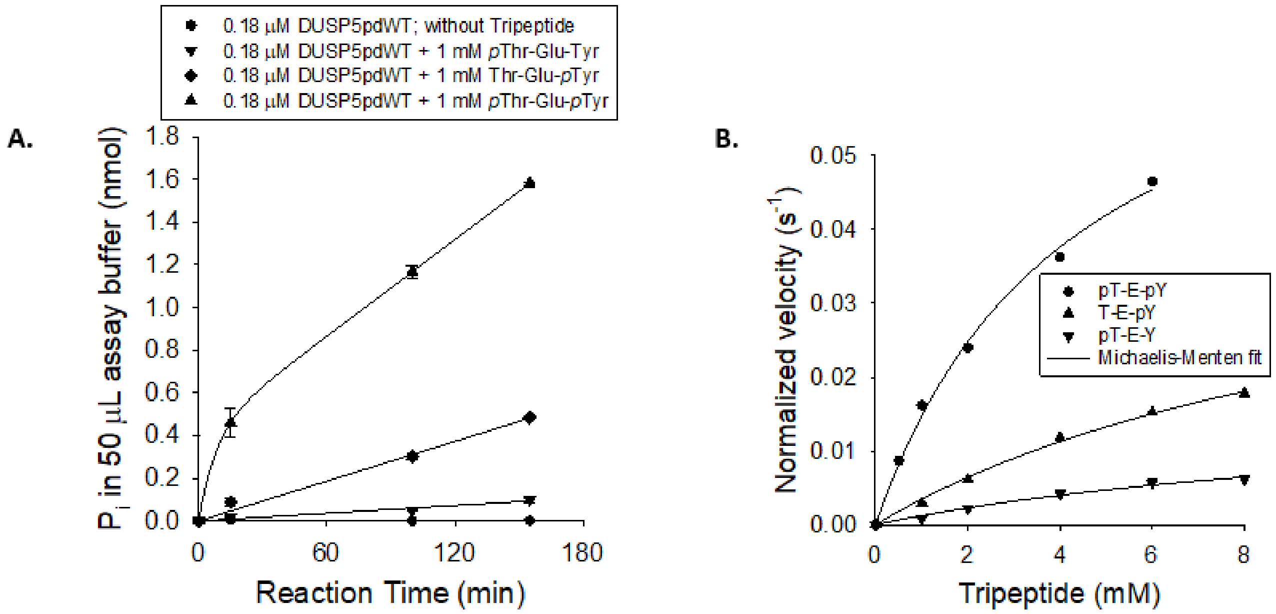

Fig. S6. (A) Time course of inorganic phosphate (P_i) generation from mono- and di- phosphorylated tripeptide in the presence of DUSP5pdWT (P_i measured with Biomol Green reagent). (B) Michaelis-Menten curves for phosphorylated ERK2 activation loop tripeptides as substrates for DUSP5pdWT, with phosphate detected using Biomol Green reagent. Plots in panel B reflects a nonlinear least squares fit.

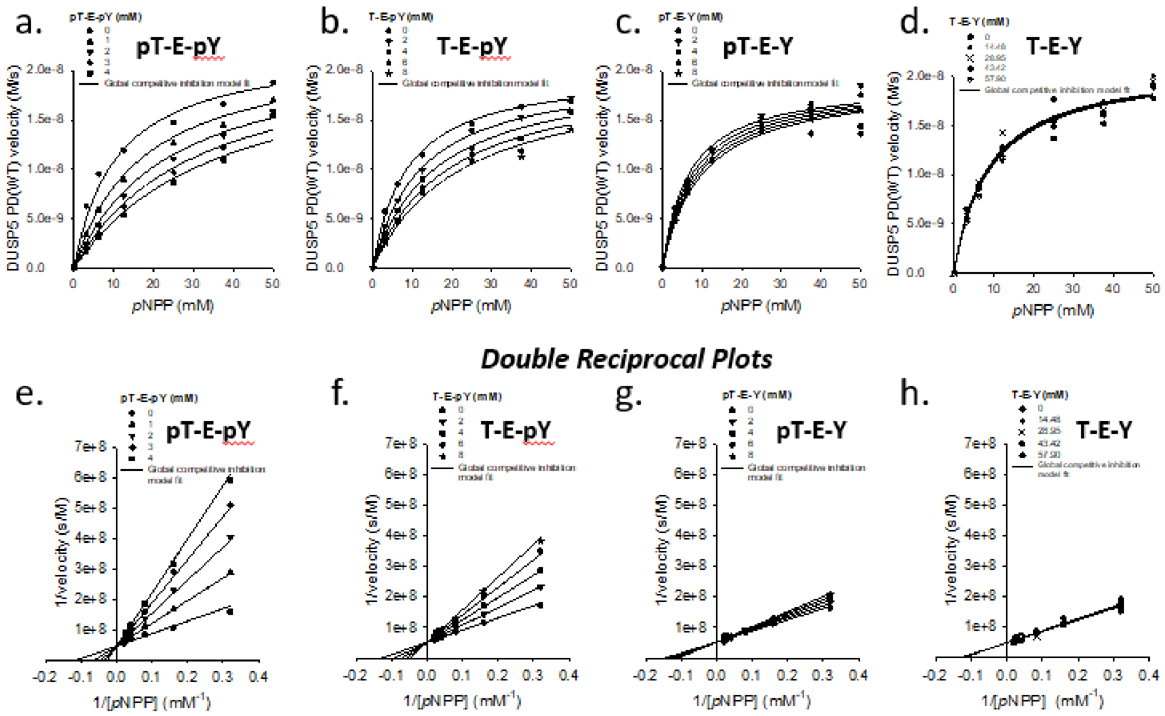

Fig. S7. Full Michaelis-Menten global competitive inhibition model fits for ERK2 activation loop tripeptides in various phosphorylation states (see Table S2). Plots reflect nonlinear least squares fits.

A.

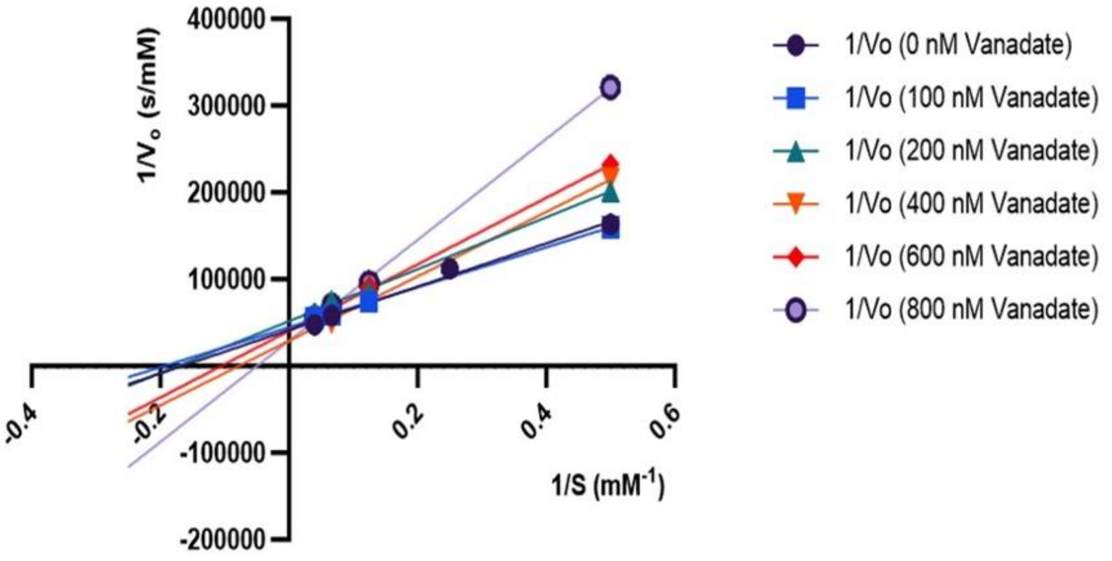

B.

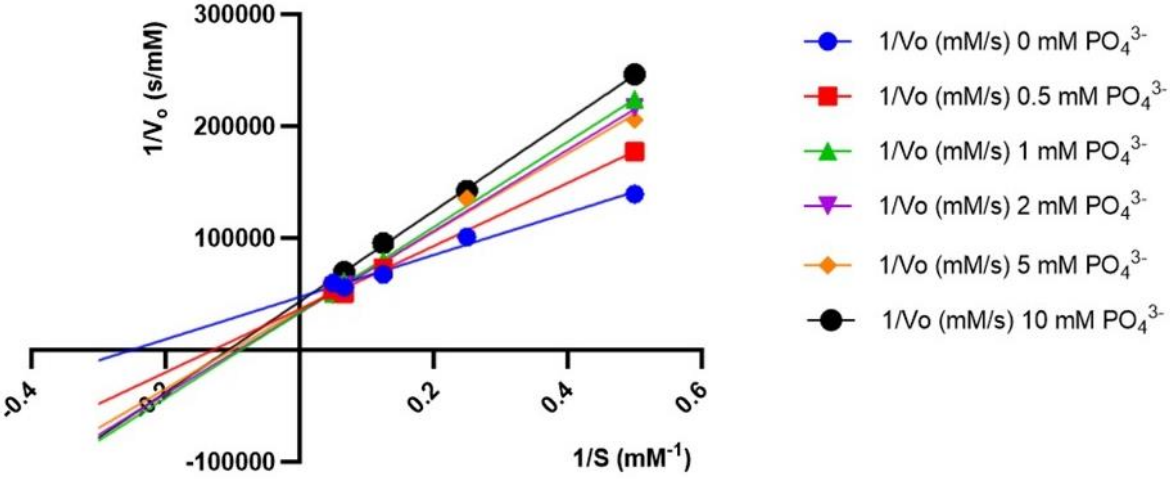

Fig. S8. (A) Lineweaver Burk plot of DUSP5pdWT inhibition by vanadate. (B) Lineweaver Burk plot of DUSP5pdWT inhibition by phosphate.

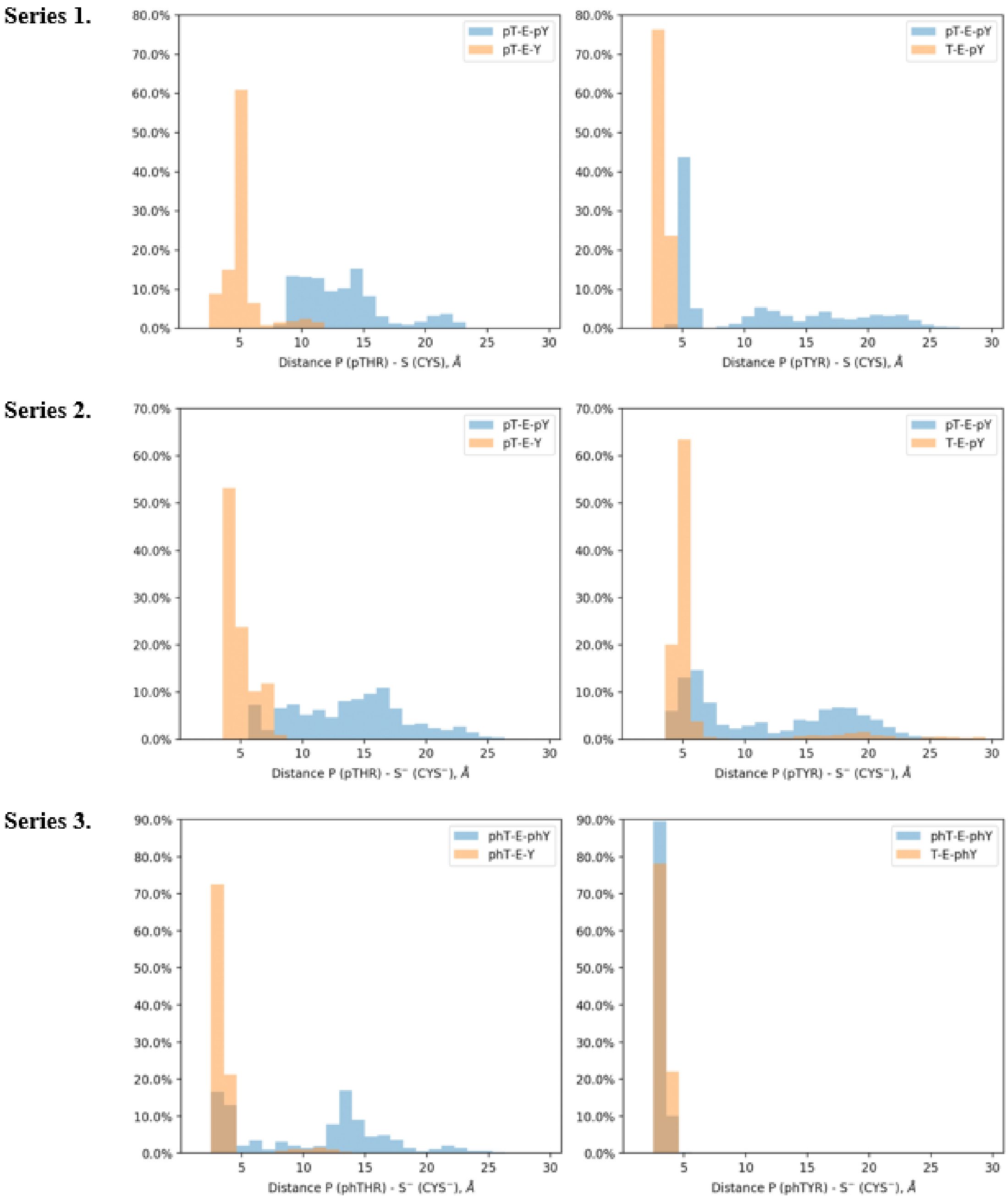

Fig. S9. Active site analysis. Distribution of distances between cysteine residue of the main active site in DUSP5 protein and phosphorylated threonine/tyrosine residues obtained by molecular dynamics simulations. Considered interactions and ionization states: **Series 1** – protonated cysteine and dianion phosphates, **Series 2** – deprotonated cysteine and dianion phosphates, **Series 3** - deprotonated cysteine and monoanion phosphates.

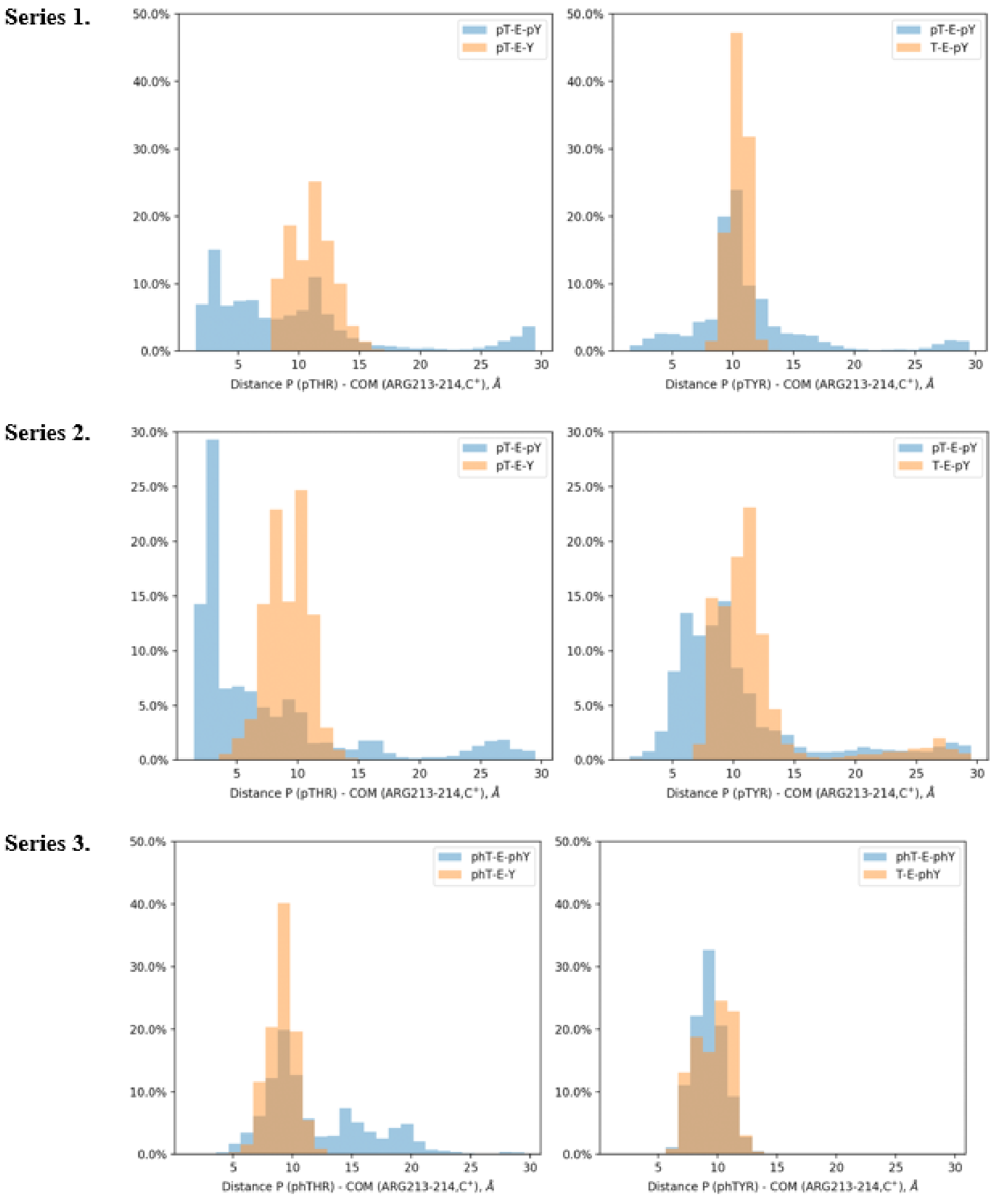

Fig. S10. Secondary site analysis. Distribution of distances between center of mass (COM) of the arginines 213, 214 of the secondary active site in DUSP5 protein and phosphorylated threonine/tyrosine residues obtained by molecular dynamics simulations and ionization states. Features of the structures: **Series 1** – protonated cysteine in the main active site and dianion phosphates, **Series 2** – deprotonated cysteine in the main active site and dianion phosphates, **Series 3 -** deprotonated cysteine in the main active site and monoanion phosphates.

Table S1: Chemical Shift Assignments for the Cleaved DUSP5pdRDB Construct

Residue Number	Identity	Atom	Chemical Shift	Residue Number	Identity	Atom	Chemical Shift
174	G	N	999.000	222	H	C	174.416
174	G	NH	999.000	222	H	CA	56.166
174	G	CA	999.000	222	H	CB	28.731
174	G	CB	999.000	223	L	HN	7.586
174	G	C	999.000	223	L	N	120.134
175	S	C	999.000	223	L	C	180.010
175	S	N	999.000	223	L	CA	53.431
175	S	HN	999.000	223	L	CB	42.736
175	S	CA	999.000	224	H	HN	8.027
175	S	CB	64.613	224	H	N	119.986
176	H	C	174.800	224	H	C	174.458
176	H	CA	55.980	224	H	CA	56.131
176	H	CB	64.610	224	H	CB	30.554
176	H	N	121.148	225	Y	HN	8.921
176	H	HN	8.716	225	Y	N	125.809
176	H	CB	29.726	225	Y	C	173.881
177	M	C	176.490	225	Y	CA	57.846
177	M	CA	55.890	225	Y	CB	43.316
177	M	CB	64.610	226	K	HN	7.975
177	M	N	122.036	226	K	N	128.045
177	M	HN	8.506	226	K	C	171.518
178	G	CA	44.550	226	K	CA	54.862
178	G	C	999.000	226	K	CB	33.869
178	G	N	109.258	227	W	HN	8.610
178	G	HN	8.276	227	W	N	129.162
179	P	C	174.900	227	W	C	175.363
179	P	CA	62.480	227	W	CA	53.371
179	P	N	999.000	227	W	HE	9.555
179	P	HN	999.000	227	W	CB	31.714
179	P	CB	33.050	228	I	HN	8.946
180	V	C	174.620	228	I	N	128.185
180	V	CA	60.460	228	I	C	999.000
180	V	N	116.548	228	I	CA	58.011
180	V	HN	8.579	229	P	HN	999.000
180	V	CB	35.029	229	P	N	999.000
181	E	C	175.180	229	P	C	175.846
181	E	CA	57.390	229	P	CA	61.404
181	E	N	128.286	229	P	CB	30.057
181	E	HN	8.709	230	V	HN	6.963

182	I	C	175.210	230	V	N	123.106
182	I	CA	60.900	230	V	C	174.284
182	I	N	128.690	230	V	CA	56.243
182	I	HN	8.82	230	V	CB	35.526
182	I	CB	36.604	231	E	HN	8.647
183	L	C	999.000	231	E	N	126.534
183	L	CA	52.920	231	E	C	175.843
183	L	N	119.147	231	E	CB	31.797
183	L	HN	8.234	232	D	HN	8.983
184	P	HN	999.000	232	D	N	122.346
184	P	N	999.000	232	D	C	175.099
184	P	CA	999.000	232	D	CA	52.660
184	P	C	999.000	232	D	CB	39.255
185	F	HN	5.691	233	S	HN	7.193
185	F	N	104.402	233	S	N	115.562
185	F	C	173.430	233	S	CA	55.111
185	F	CA	999.000	233	S	C	999.000
185	F	CB	40.167	234	H	HN	999.000
186	L	HN	7.696	234	H	N	999.000
186	L	N	126.469	234	H	C	174.525
186	L	C	171.771	234	H	CA	57.706
186	L	CA	999.000	234	H	CB	28.565
186	L	CB	47.045	235	T	HN	7.252
187	Y	HN	9.597	235	T	N	107.379
187	Y	N	127.738	235	T	C	174.625
187	Y	C	173.270	235	T	CA	61.141
187	Y	CA	56.690	235	T	CB	69.088
187	Y	CB	43.067	236	A	HN	7.323
188	L	HN	9.018	236	A	N	126.659
188	L	N	123.301	236	A	C	176.876
188	L	C	175.646	236	A	CA	52.666
188	L	CA	53.160	236	A	CB	21.290
188	L	CB	46.548	237	D	HN	8.595
189	G	HN	8.405	237	D	N	121.636
189	G	N	111.885	237	D	C	176.073
189	G	C	170.783	237	D	CA	53.124
189	G	CA	46.190	237	D	CB	40.664
190	S	HN	10.038	238	I	HN	8.582
190	S	N	118.476	238	I	N	125.048
190	S	C	175.312	238	I	C	175.759
190	S	CA	56.240	238	I	CA	62.675
190	S	CB	67.928	238	I	CB	40.996
191	A	HN	8.040	239	S	HN	8.072

191	A	N	122.549	239	S	N	119.434
191	A	C	180.001	239	S	C	178.025
191	A	CA	55.320	239	S	CA	58.246
191	A	CB	18.787	239	S	CB	63.453
192	Y	HN	8.114	240	S	HN	8.330
192	Y	N	119.129	240	S	N	119.544
192	Y	C	175.631	240	S	C	174.686
192	Y	CA	68.880	240	S	CA	61.058
192	Y	CB	38.178	240	S	CB	62.044
193	H	HN	7.145	241	H	HN	7.667
193	H	N	118.835	241	H	N	116.260
193	H	C	999.000	241	H	C	174.793
193	H	CA	52.970	241	H	CA	54.740
194	A	HN	999.000	241	H	CB	29.063
194	A	N	999.000	242	F	HN	7.487
194	A	C	176.260	242	F	N	119.612
194	A	CA	59.020	242	F	C	177.281
194	A	CB	18.041	242	F	CA	58.011
195	C	HN	7.269	242	F	CB	35.858
195	C	N	109.589	243	Q	HN	8.940
195	C	C	173.988	243	Q	N	119.840
195	C	CA	54.060	243	Q	C	177.606
196	K	HN	7.009	243	Q	CA	59.517
196	K	N	122.097	243	Q	CB	27.571
196	K	C	176.060	244	E	HN	8.519
196	K	CA	62.560	244	E	N	118.546
196	K	CB	32.377	244	E	C	177.468
197	C	HN	8.579	244	E	CA	58.900
197	C	N	123.654	244	E	CB	29.477
197	C	C	176.298	245	A	HN	7.928
197	C	CA	59.900	245	A	N	122.830
198	E	HN	9.402	245	A	C	178.342
198	E	N	120.317	245	A	CA	55.665
198	E	C	178.527	245	A	CB	19.782
198	E	CA	59.060	246	I	HN	8.767
198	E	CB	29.228	246	I	N	120.081
199	F	HN	7.052	246	I	C	177.341
199	F	N	116.508	246	I	CA	999.000
199	F	C	177.690	247	D	HN	9.045
199	F	CA	57.400	247	D	N	118.422
199	F	CB	38.592	247	D	C	179.096
200	L	HN	7.151	247	D	CA	57.745
200	L	N	116.086	247	D	CB	40.498

200	L	C	179.231	248	F	HN	7.626
200	L	CA	54.860	248	F	N	120.842
200	L	CB	41.161	248	F	C	176.648
201	A	HN	8.342	248	F	CA	62.021
201	A	N	120.514	249	I	HN	8.344
201	A	CA	54.856	249	I	N	119.256
201	A	C	181.284	249	I	C	177.711
202	N	HN	7.864	249	I	CA	66.335
202	N	N	116.059	249	I	CB	38.344
202	N	CA	55.819	250	D	HN	8.881
202	N	CB	38.510	250	D	N	117.945
202	N	C	176.699	250	D	C	178.350
203	L	HN	7.079	250	D	CA	57.976
203	L	N	115.701	250	D	CB	40.747
203	L	CA	54.432	251	C	HN	8.038
203	L	C	176.079	251	C	N	120.720
203	L	CB	42.819	251	C	C	177.170
204	H	HN	7.474	251	C	CA	62.560
204	H	N	114.270	251	C	CB	26.245
204	H	C	173.767	252	V	HN	7.390
204	H	CA	56.474	252	V	N	120.321
204	H	CB	25.914	252	V	C	178.744
205	I	HN	7.842	252	V	CA	66.296
205	I	N	117.061	252	V	CB	31.300
205	I	C	176.234	253	R	HN	8.417
205	I	CA	61.019	253	R	N	120.993
205	I	CB	37.349	253	R	C	999.000
206	T	HN	8.798	253	R	CA	999.000
206	T	N	115.733	253	R	CB	30.233
206	T	C	174.872	254	E	HN	8.287
206	T	CA	61.906	254	E	N	119.931
206	T	CB	70.414	254	E	C	177.752
207	A	HN	8.120	254	E	CA	59.093
207	A	N	125.625	254	E	CB	29.477
207	A	C	174.536	255	K	HN	7.254
207	A	CA	51.273	255	K	N	116.493
207	A	CB	25.334	255	K	C	177.160
208	L	HN	8.955	255	K	CA	55.780
208	L	N	121.402	255	K	CB	32.543
208	L	C	173.663	256	G	HN	7.934
208	L	CA	53.848	256	G	N	108.337
208	L	CB	47.128	256	G	C	175.288
209	L	HN	8.828	256	G	CA	46.112

209	L	N	124.667	257	G	HN	7.820
209	L	C	172.836	257	G	N	107.296
209	L	CA	53.244	257	G	C	172.837
209	L	CB	44.559	257	G	CA	44.421
210	N	HN	8.402	258	K	HN	7.762
210	N	N	124.027	258	K	N	114.306
210	N	C	174.481	258	K	C	174.702
210	N	CA	51.299	258	K	CA	56.243
210	N	CB	40.416	258	K	CB	38.591
211	V	HN	8.633	259	V	HN	8.983
211	V	N	127.728	259	V	N	122.005
211	V	C	173.457	259	V	C	173.347
211	V	CA	59.247	259	V	CA	58.301
211	V	CB	30.306	259	V	CB	35.941
212	S	HN	7.454	260	L	HN	8.514
212	S	N	113.042	260	L	N	127.660
212	S	C	174.177	260	L	C	174.148
212	S	CA	58.477	260	L	CA	53.536
212	S	CB	65.193	260	L	CB	44.062
213	R	HN	8.665	261	V	HN	9.323
213	R	N	118.454	261	V	N	130.867
213	R	C	175.419	261	V	C	174.531
213	R	CA	57.051	261	V	CA	61054.000
214	R	HN	8.292	261	V	CB	32.129
214	R	N	120.789	262	H	HN	9.240
214	R	C	175.016	262	H	N	123.538
214	R	CA	55.511	262	H	C	999.000
214	R	CB	32.460	262	H	CA	53.909
215	T	HN	8.033	263	S	HN	999.000
215	T	N	117.418	263	S	N	999.000
215	T	C	173.638	263	S	C	174.797
215	T	CA	61.019	263	S	CA	56.666
216	S	HN	7.421	264	E	HN	7.932
216	S	N	117.985	264	E	N	121.191
216	S	C	174.364	264	E	C	177.726
216	S	CA	58.199	264	E	CA	61.366
216	S	CB	30.223	264	E	CB	30.223
217	E	HN	8.349	265	A	HN	9.885
217	E	N	123.210	265	A	N	119.457
217	E	C	176.307	265	A	C	179.489
217	E	CA	56.751	265	A	CA	52.352
217	E	CB	30.223	265	A	CB	20.527
218	A	HN	8.006	266	G	HN	7.973

218	A	N	123.962	266	G	N	109.029
218	A	C	179.115	266	G	C	173.723
218	A	CA	52.776	266	G	CA	47.075
218	A	CB	10.616	267	I	HN	9.386
219	C	HN	8.135	267	I	N	118.276
219	C	N	117.199	267	I	C	176.162
219	C	C	174.844	267	I	CA	63.215
219	C	CA	58.977	267	I	CB	41.576
219	C	CB	27.820	268	S	HN	9.736
220	M	HN	7.858	268	S	N	117.732
220	M	N	121.651	268	S	C	999.000
220	M	C	176.265	268	S	CA	59.171
220	M	CA	55.549	269	R	HN	12.091
220	M	CB	33.869	269	R	N	127.091
221	T	HN	7.842	269	R	C	177.955
221	T	N	113.347	269	R	CA	61.712
221	T	C	177.319	270	S	HN	7.667
221	T	CA	63.292	270	S	N	106.877
222	H	HN	8.436	270	S	C	999.000
222	H	N	116.611	270	S	CA	62.776

Note: a chemical shift of 999.0

Table S2. Comparison of kinetic parameters for DUSP5pdWT hydrolysis of para-nitrophenyl phosphate (*pNPP*) in the presence of pERK tripeptide mimetics as inhibitors, and having different phosphorylation states. Kinetic parameters were calculated from global non-linear regression competitive inhibition model fits (Michaelis-Menten) to equation S1 and global linear regression competitive inhibition model fits (Lineweaver-Burk) to equation S2.

Global Competitive Enzyme Inhibition Model: Michaelis-Menten Fits				
Kinetic Parameters	*Peptides*			
	pT-E-pY	T-E-pY	pT-E-Y	T-E-Y
k_{cat} (s^{-1})	$2.03 \pm 0.07 \times 10^{-2}$	$1.82 \pm 0.04 \times 10^{-2}$	$1.71 \pm 0.04 \times 10^{-2}$	$1.92 \pm 0.05 \times 10^{-2}$
K_m (mM)	10.77 ± 1.21	8.56 ± 0.68	6.50 ± 0.68	8.41 ± 0.90
K_i (mM)	1.67 ± 0.20	4.93 ± 0.55	15.76 ± 5.24	583 ± 764
k_{cat}/K_m ($M^{-1}s^{-1}$)	1.88	2.13	2.63	2.28
R^2	0.99	0.99	0.98	0.98
Sy.x	7.16×10^{-10}	5.31×10^{-10}	7.90×10^{-10}	9.55×10^{-10}
Global Competitive Inhibition Model: Lineweaver-Burk Fits				
k_{cat} (s^{-1})	$1.98 \pm 0.10 \times 10^{-2}$	$1.75 \pm 0.04 \times 10^{-2}$	$1.70 \pm 0.04 \times 10^{-2}$	$1.84 \pm 0.08 \times 10^{-2}$
K_m (mM)	8.99 ± 0.79	7.72 ± 0.37	6.79 ± 0.29	7.58 ± 0.64
K_i (mM)	1.22 ± 0.08	4.80 ± 0.24	21.93 ± 2.83	913 ± 1125
k_{cat}/K_m ($M^{-1}s^{-1}$)	2.20	2.27	2.50	2.43
R^2	1.00*	1.00*	0.99	0.96
Sy.x	8.72×10^{6}	4.92×10^{6}	4.18×10^{6}	8.29×10^{6}

* Values were rounded. Actual R^2 was 0.997.

$$v = \frac{V_{max}[S]}{K_m\left(1+\frac{[I]}{K_i}\right)+[S]} \qquad \textit{(equation S1)}$$

$$\frac{1}{v} = \left((K_m/k_{cat}) \times \left(1 + (I/K_i)\right)\right) \times [S] + 1/k_{cat} \qquad \textit{(equation S2)}$$
